# Extracellular Vesicle Secretion by Leukemia Cells *In Vivo* Promotes CLL Progression by Hampering Antitumor T-cell Responses

**DOI:** 10.1158/2643-3230.BCD-22-0029

**Published:** 2022-09-14

**Authors:** Ernesto Gargiulo, Elodie Viry, Pablo Elías Morande, Anne Largeot, Susanne Gonder, Feng Xian, Nikolaos Ioannou, Mohaned Benzarti, Felix Bruno Kleine Borgmann, Michel Mittelbronn, Gunnar Dittmar, Petr V. Nazarov, Johannes Meiser, Basile Stamatopoulos, Alan G. Ramsay, Etienne Moussay, Jérôme Paggetti

**Affiliations:** 1 Tumor–Stroma Interactions Group, Department of Cancer Research, Luxembourg Institute of Health, Luxembourg City, Luxembourg.; 2 Instituto de Medicina Experimental (IMEX)-CONICET-Academia Nacional de Medicina, Buenos Aires, Argentina.; 3 Faculty of Science, Technology and Medicine, University of Luxembourg, Esch-sur-Alzette, Luxembourg.; 4 Proteomics of Cellular Signaling, Department of Infection and Immunity, Luxembourg Institute of Health, Strassen, Luxembourg.; 5 School of Cancer and Pharmaceutical Sciences, Faculty of Life Sciences and Medicine, King's College London, London, United Kingdom.; 6 Cancer Metabolism Group, Department of Cancer Research, Luxembourg Institute of Health, Luxembourg City, Luxembourg.; 7 Department of Neurosurgery, Centre Hospitalier de Luxembourg, Luxembourg City, Luxembourg.; 8 Luxembourg Centre of Neuropathology, Department of Cancer Research, Luxembourg Institute of Health, Luxembourg City, Luxembourg.; 9 Luxembourg Centre of Neuropathology, University of Luxembourg, Esch-sur-Alzette, Luxembourg.; 10 Department of Life Sciences and Medicine, University of Luxembourg, Esch-sur-Alzette, Luxembourg.; 11 National Center of Pathology, Laboratoire national de santé (LNS), Dudelange, Luxembourg.; 12 Luxembourg Centre for Systems Biomedicine, University of Luxembourg, Esch-sur-Alzette, Luxembourg.; 13 Multiomics Data Science Group, Department of Cancer Research, Luxembourg Institute of Health, Strassen, Luxembourg.; 14 Labora­tory of Clinical Cell Therapy, Jules Bordet Institute, Université Libre de Bruxelles, Brussels, Belgium.

## Abstract

Small extracellular vesicle (sEV, or exosome) communication among cells in the tumor microenvironment has been modeled mainly in cell culture, whereas their relevance in cancer pathogenesis and progression *in vivo* is less characterized. Here we investigated cancer–microenvironment interactions *in vivo* using mouse models of chronic lymphocytic leukemia (CLL). sEVs isolated directly from CLL tissue were enriched in specific miRNA and immune-checkpoint ligands. Distinct molecular components of tumor-derived sEVs altered CD8^+^ T-cell transcriptome, proteome, and metabolome, leading to decreased functions and cell exhaustion *ex vivo* and *in vivo*. Using antagomiRs and blocking antibodies, we defined specific cargo-mediated alterations on CD8^+^ T cells. Abrogating sEV biogenesis by *Rab27a/b* knockout dramatically delayed CLL pathogenesis. This phenotype was rescued by exogenous leukemic sEV or CD8^+^ T-cell depletion. Finally, high expression of sEV-related genes correlated with poor outcomes in CLL patients, suggesting sEV profiling as a prognostic tool. In conclusion, sEVs shape the immune microenvironment during CLL progression.

**Significance:**

sEVs produced in the leukemia microenvironment impair CD8^+^ T-cell mediated antitumor immune response and are indispensable for leukemia progression *in vivo* in murine preclinical models. In addition, high expression of sEV-related genes correlated with poor survival and unfavorable clinical parameters in CLL patients.

*See related commentary by Zhong and Guo, p. 5.*

*This article is highlighted in the In This Issue feature, p. 1*

## Teaser

Exosomes produced in the leukemia microenvironment inhibit CD8^+^ T cell-mediated antitumor immunity by immune checkpoint interactions and miRNA transfer sustaining CLL development in mouse models.

## INTRODUCTION

Small extracellular vesicles (sEV), or exosomes, are small-size particles (30–150 nm) released by every cell and found in any body fluid. They are involved in cell-to-cell communication through the transfer of genetic material and proteins (1), and ligands/receptors interactions, affecting biological functions of target cells (2). Release of sEVs and their cargo depends on the cellular and physiologic context (3). Tumor-derived sEVs are involved in the reeducation of microenvironment cells promoting tumor proliferation, immune escape, and metastasis (4–7).

The survival and proliferation of chronic lymphocytic leukemia (CLL) cells depend strictly on interactions with the microenvironment (8, 9). CLL cells evolve in a highly immunosuppressive environment (10) where sEVs affect progression, invasion, and resistance to treatment (7). We and others previously demonstrated that CLL-derived sEVs reeducate surrounding cells and enhance immune-escape mechanisms (11–13), such as stromal cell conversion into cancer-associated fibroblasts and modulation of PD-L1 expression on monocytes, among others (14, 15).

However, these studies are mostly based on *in vitro*–generated sEVs. In light of the complex leukemia microenvironment (LME) composition, the role of sEVs in CLL pathogenesis *in vivo* still needs to be evaluated. Herein, we developed a protocol to isolate sEVs directly from the LME of Eμ-*TCL1* mice with the aim to uncover their complexity and their role in CLL development and progression *in vivo*. We show that sEVs isolated from the LME contain specific miRNA and proteins and display on their surface multiple immune-checkpoints (ICP), which play a key role in CLL development, by hampering the antitumor CD8^+^ T cell–mediated immune response. LME-sEVs reprogrammed the CD8^+^ T-cell transcriptome, proteome, and metabolome, leading to cell exhaustion, decreased granzyme B production, cytokine secretion, and tumor cell lysis. Decrease of sEV secretion by *Rab27* KO led to the impairment of CLL growth *in vivo*. Finally, we showed in a cohort of CLL patients that the expression of genes involved in sEV biogenesis and secretion differs between groups defined by the classic prognosis markers and correlates with survival. Overall, our findings highlight the importance of sEVs in the microenvironment for leukemia development and progression.

## RESULTS

### sEVs Are Enriched in Leukemia Microenvironment

**Figure 1.**
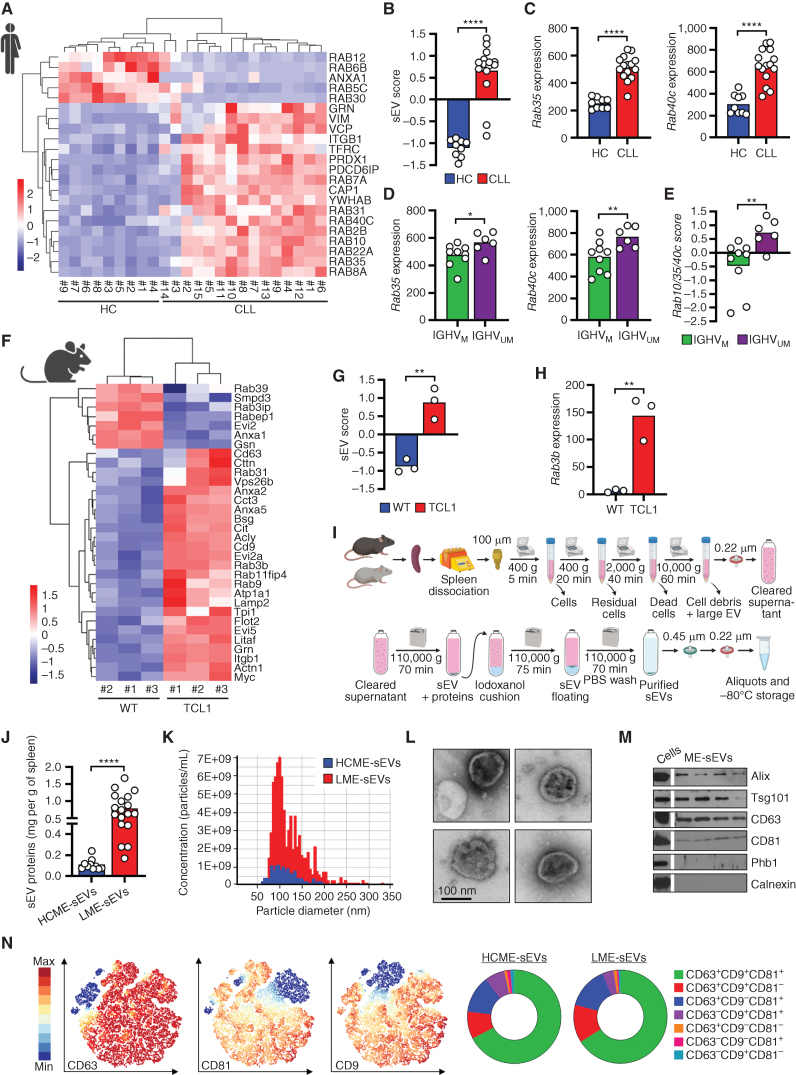
sEV are enriched in the human and murine leukemic microenvironments. **A,** Relative mRNA expression of selected genes involved in sEV biogenesis and secretion in B cells from PB of healthy donors (HC, *n* = 9) and CLL patients (CLL, *n* = 15; from GSE67640). **B,** Score based on sEV-related mRNA levels from **A**. **C,** mRNA levels of selected genes extracted from **A**. **D,** mRNA expression of selected genes according to IGHV mutational status. **E,** Score combining the expression of *Rab10*, *Rab35*, and *Rab40C* according to IGHV mutational status. **F,** Relative mRNA expression of selected genes involved in sEV biogenesis and secretion in B cells from C57BL/6 (WT) and Eμ-TCL1 mice (TCL1; from GSE175564). **G,** Score based on sEV-related RNA levels from **F**. **H,***Rab3b* mRNA level extracted from **F**. **I,** Detailed protocol to isolate and purify sEVs from the murine spleen. **J,** Amount of proteins (in mg) recovered from LME- (*n* = 18) or HCME-sEVs (*n* = 10), normalized per gram of spleen. **K,** Representative TRPS analysis of ME-sEVs for size and concentration. **L,** Electron microscopy images of ME-sEVs. **M,** Western blot analysis of ME-sEVs. **N,** HSNE clustering analysis of MB488^+^ LME-sEVs based on CD63, CD81, and CD9 expression measured by bead-free FC (left) and relative percentages of combined expression (right). *, *P* < 0.05; **, *P* < 0.01; ****, *P* < 0.0001 (unpaired Student *t* test). Data are mean.

To assess the relevance of sEV biogenesis in CLL, we performed gene-expression analysis using a public data set (16) comparing CLL patient cells with normal B cells. We observed higher expression of genes associated with sEV biogenesis in CLL cells (Fig. 1A), whereas regulators of retrograde transport and lysosomal degradation were less expressed, resulting in higher computed sEV scores (Fig. 1B). Furthermore, we observed a higher expression of typical sEV markers, such as the RAB family members and the programmed cell death 6-interacting protein (PDCD6IP, also known as ALIX), associated with increased sEV production (Fig. 1C; Supplementary Fig. S1A; refs. 17–19). This is in accordance with increased levels of CLL-derived sEVs (CD20^+^) in the plasma of CLL patients compared with healthy controls, as we previously reported (15, 20). Interestingly, individual or combined gene expression of major sEV biogenesis regulators, such as *Rab10*, *Rab35*, and *Rab40*c, was higher in cells from patients with unfavorable UM-IGHV than from patients with M-IGHV (Fig. 1D and E; Supplementary Fig. S1B). Similar results were obtained with the Eμ-*TCL1* (TCL1) CLL murine model (GSE175564, ref. 21; vs. WT B cells from C57BL/6 mouse; Fig. 1F and G) as *Cd9*, *Rab3b*, and *Rab31* were upregulated (Fig. 1H; Supplementary Fig. S1C) in leukemic cells, suggesting an important role of sEVs in CLL pathology (18, 22, 23).

Commonly, sEVs are isolated from cell line culture supernatants, allowing to conveniently isolate tumor-derived sEVs in large amounts and study their functional impact on accessory cells. However, this method does not fully reflect the complexity of a whole organism. As sEV biogenesis and release are dynamic processes, we aimed to obtain a closer biological representation of sEVs from the CLL microenvironment (24). Therefore, we established a protocol to isolate sEV directly from the spleen of murine models (Fig. 1I), preserving at the same time cell integrity (Supplementary Fig. S1D). First, we detected a 10-fold enrichment in LME-sEVs compared with healthy controls (HCME-sEV; Fig. 1J). Similarly, we found greater levels of circulating sEVs in the peripheral blood (PB) of TCL1 mice compared with healthy controls (Supplementary Fig. S1E), further highlighting sEV potential impact in leukemogenesis.

Overall, isolated ME-sEVs showed expected size and morphology (80–120 nm; Fig. 1K and L), presence of typical sEV markers (Fig. 1M; Supplementary Fig. S1F), and the absence of major contaminants for both conditions, validating their use for further downstream analysis. In order to analyze more in-depth the tetraspanin distribution on ME-sEVs from both sources, we performed single sEV FC analysis of CD63, CD81, and CD9 (Fig. 1N). Phenotyping of single sEV suggests alteration of tetraspanins distribution between LME- and HCME-sEVs. Hierarchical clustering further highlighted the heterogeneity of the tetraspanin distribution on single sEV, showing different combinations of CD63, CD9, and CD81 within ME-sEVs subsets (Supplementary Fig. S1G). Interestingly, tetraspanin distribution analysis reveals the expansion of distinct LME-sEVs subpopulations compared with the healthy counterpart (clusters C1–C10; Supplementary Fig. S1H). These observations, beyond emphasizing the risk of introducing a bias when isolating sEVs with single marker-directed beads, highlight the heterogeneity of tumor sEVs even on such widely distributed markers.

### LME-sEVs Present a Specific Proteome and miRNA Fingerprint

**Figure 2.**
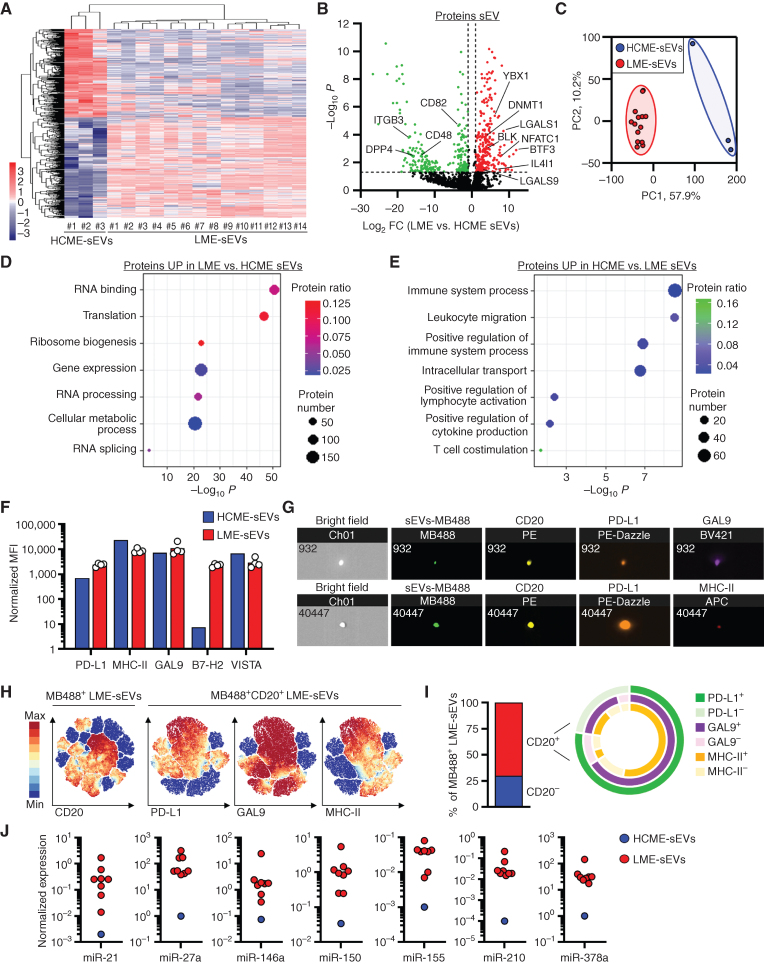
LME-sEVs present a specific proteome and miRNA fingerprint. **A,** Hierarchical clustering of sEVs differentially expressed proteins (DEP with *q* < 0.05) identified by mass spectrometry between HCME-sEVs (*n* = 3, isolated from independent pools of 5 C57BL/6 spleens) and LME-sEVs (*n* = 14). **B,** Volcano plot showing DEP between LME- and HCME-sEVs with FDR <0.05 and log_2_FC >1. **C,** PCA based on DEP. **D** and **E,** Ontology analysis of DEP between LME-sEVs and HCME-sEVs. **F,** Expression of ICP ligands on HCME- or LME-sEVs quantified by bead-based FC. **G,** Representative pictures of ICP ligand expression on single MB488^+^CD20^+^ LME-sEVs visualized by imaging FC. **H** and **I,** HSNE clustering of MB488^+^ LME-sEVs based on CD20, PD-L1, GAL9, and MHC-II expression analyzed by FC and related combinations of markers on CD20^+^ LME-sEVs. **J,** miRNA levels measured using RT-qPCR from HCME- (isolated from a pool of 5 C57BL/6 mice spleens) and LME-sEVs (*n* = 8). Data are mean.

Although we observed a 10-fold enrichment in sEVs in the LME compared with HCME, as well as an alteration of the tetraspanins distribution, we investigated whether their protein and miRNA cargo composition differed. First, a label-free proteomic analysis of equal amounts of proteins derived from LME-sEVs and HCME-sEVs identified 1,865 proteins with 527 proteins differentially present between groups (Fig. 2A–C; Supplementary Table S1). Proteins more abundant in LME-sEVs (*n* = 281) were involved in translation, RNA splicing, and binding, and in the regulation of gene expression and cellular metabolic processes (Fig. 2D), whereas proteins depleted (*n* = 246) were associated with intracellular transport and with the positive regulation of immune processes, more specifically in lymphocyte activation, migration, and cytokine production (Fig. 2E), suggesting that LME-sEVs may not optimally sustain immune responses but rather stimulate leukemic growth. Given that our proteomic analysis suggested the presence of some conventional (LGALS1 and LGALS9) and metabolic (IL4I1) ICP in LME-sEV (Fig. 2B), we analyzed the presence of further immune regulatory proteins on the ME-sEV surface. Interestingly, LME-sEVs carried several ICP ligands, including PD-L1, GAL9, B7-H2, VISTA, and MHC I/II (Fig. 2F; Supplementary Fig. S2A and S2B). Furthermore, imaging FC highlighted the coexpression of multiple ICP ligands on CD20^+^ CLL-derived sEVs (Fig. 2G). The latter was confirmed with single-sEV analysis combined with hierarchical stochastic neighbor embedding (HSNE) clustering, showing PD-L1 and GAL9 often coexpressed on CD20^+^MHC-II^+^ vesicles (Fig. 2H–I), suggesting LME-sEV immunosuppressive capabilities toward immune cells expressing corresponding receptors. We also observed surface expression of the ectonucleotidases CD39 and CD73, capable to hydrolyze surrounding ATP into adenosine, on LME-sEVs (Supplementary Fig. S2C).

We previously reported several miRNA enriched in the plasma of CLL patients (e.g., miR-150 and -155) that correlated with unfavorable clinical parameters (24), and also showed their transfer and functional activity into target cells (14, 25). Therefore, we screened a shortlist of miRNA known to be abundant in CLL and other cancers. Our data showed that murine LME-sEVs abundantly carry several miRNA, such as miR-150, -155, -21, -146a, -378a, and -27a (Fig. 2J), typically found in sEVs isolated from MEC-1 culture supernatant and patient plasma (Supplementary Fig. S2D; ref. 15). Although not previously reported in CLL-derived sEVs, miR-210, a miRNA associated with hypoxia, was found highly abundant and enriched in LME-sEVs, highlighting the dependency of sEV cargo on microenvironment conditions, which are not reflected in cell culture *in vitro*.

### LME-sEVs Enter Different Lymphocyte Subsets and Modify CD8^+^ T Cells in the Microenvironment

**Figure 3.**
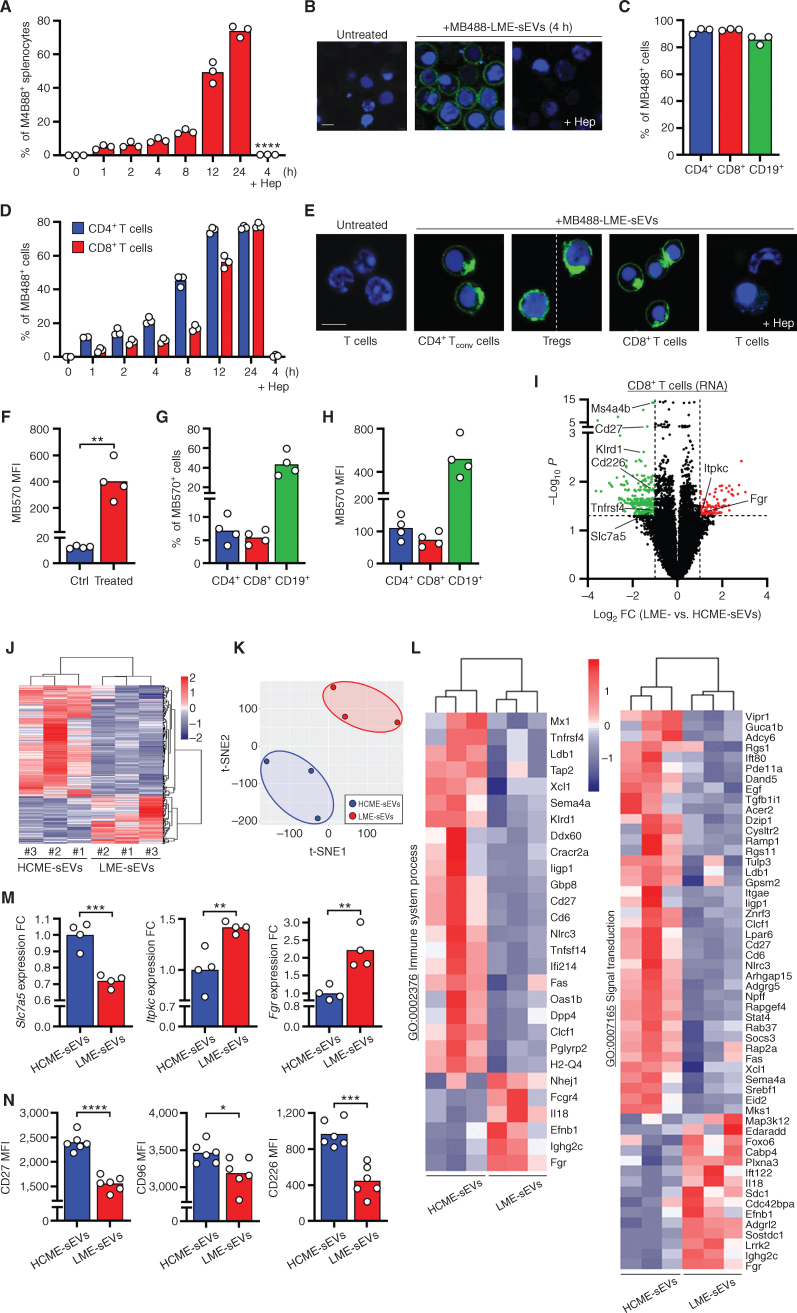
LME-sEVs enter different lymphocyte subsets and modify CD8^+^ T cells in the microenvironment. **A,** Percentage of splenocytes internalizing MB488^+^ sEVs. Splenocytes from C57BL/6 mice were incubated for increasing periods of time with MB488^+^ LME-sEVs and analyzed by FC. sEV preincubation with heparin sulfate (Hep) was performed for 4 hours. **B,** Representative confocal microscopy pictures of total splenocytes after 4 hours of treatment with LME-sEVs, in the absence or presence of heparin (scale bar, 5 μm). **C,** Splenocytes from C57BL/6 mice were incubated for 24 hours with MB488^+^ LME-sEVs and then analyzed by FC with lymphocyte-lineage markers. **D,** FACS-sorted CD4^+^ and CD8^+^ T cells were incubated for increasing periods of time with MB488^+^ LME-sEVs and analyzed by FC. **E,** Representative confocal microscopy pictures of FACS-sorted CD4^+^ T_conv_ cells, CD8^+^ T cells, and Tregs after treatment with LME-sEVs (24 hours; scale bar, 5 μm). **F–H,** MB570^+^-LME-sEVs were i.v. injected in C57BL/6 mice. Total splenocytes were harvested 24 hours later and analyzed by FC directly (**F**) or after staining for specific immune subsets (CD19^+^ B cells, CD4^+^ and CD8^+^ T cells, **G–H**). **I,** Volcano plot showing differential expression of genes (DEG) with FDR <0.05 and log_2_FC >1 in CD8^+^ T cells isolated from spleens of mice treated with LME- or HCME-sEVs for 1 week. **J,** Hierarchical clustering of DEG from **I**. **K,** t-distributed stochastic neighbor embedding (t-SNE) of samples from **I**. **L,** Hierarchical clustering of selected genes from **J**, grouped by enriched gene ontologies. **M** and **N,** mRNA (**M**) or protein levels (**N**) of 3 selected DEG from **I**, quantified by RT-qPCR or FC, in CD8^+^ T cells treated *in vitro* for 48 hours with HCME- or LME-sEVs. *, *P* < 0.05; **, *P* < 0.01; ***, *P* < 0.001; ****, *P* < 0.0001 (unpaired Student *t* test). Data are mean.

As we isolated sEVs produced in the spleen, we sought to validate the interaction between these LME-sEVs and splenic immune cells. Splenocytes treated *ex vivo* with fluorescent-LME-sEVs displayed a rapid and time-dependent internalization that was inhibited by heparin (Fig. 3A and B), a known inhibitor of sEV uptake (14). Around 90% of each lymphocyte subsets internalized sEVs after 24 hours (Fig. 3C). To rule out a possible indirect transfer between splenocytes, we presorted different T-cell subsets. Both FC and confocal microscopy confirmed that CD4^+^ T_conv_ cells, regulatory T cells (Treg), and CD8^+^ T cells actively internalized LME-sEVs *ex vivo*. A total inhibition was observed in the presence of heparin, highlighting the specificity of the internalization process (Fig. 3D and E). Injection of fluorescent-ME-sEVs in mice led to increased fluorescence in total splenocytes, confirming ME-sEV uptake *in vivo* (Fig. 3F). In particular, 5% to 10% of T cells and 40% of B cells internalized sEVs 24 hours after a single injection (Fig. 3G and H). Our observations on T-cell subsets contrast with previous studies using *in vitro*–secreted sEVs and reporting low internalization (26, 27), highlighting the importance of using sEVs produced *in vivo* and to study their uptake in a whole organism.

In light of these results, we aimed to evaluate LME-sEV activity on immune surveillance *in vivo*. We injected LME-sEVs in Foxp3^YFP/Cre^ mice daily for 7 days, then sorted YFP^+^-Treg, CD4^+^ T_conv_, CD8^+^ T, and CD19^+^ B lymphocytes and analyzed gene expression (Fig. 3I–L; Supplementary Fig. S3; Supplementary Table S2). Surprisingly, exclusively CD8^+^ T cells showed different expression profiles (Fig. 3I–K; Supplementary Fig. S3A) with downregulation of genes involved in immune response (*Cd27*, *Cd226*, and *Ms4a4b*) and amino acid transport (*Slc7a5*), whereas genes associated with inhibition of immune response (*Itpkc*) and T-cell differentiation (*Fgr*) were upregulated (Fig. 3I and 3L). K-means clustering confirmed the repression of genes regulating immune response and lymphocyte activation (Supplementary Fig. S3B). Ontology analysis also revealed significant changes in crucial biological functions, such as actin cytoskeleton organization (Fig. 3L; Supplementary Fig. S3C). Similarly, our data showed significant changes in genes associated with oxidoreductase activity, and kinase and phosphatase activities (Supplementary Fig. S3C). Importantly, we could confirm, in a separate *ex vivo* experiment, the rapid decrease in the amino acid transporter *Slc7a5*, and of the costimulatory molecules CD27, CD96, and CD226, crucial for T-cell activation. We also observed the increase in the kinases *Itpkc* and *Fgr* (Fig. 3M and N), pointing to the negative regulation of cell activation, migration, and adhesion. Despite the changes detected in CD8^+^ T cells, no significant effect was detected in Tregs, CD4^+^ T_conv_, and CD19^+^ B cells after treating mice *in vivo* (Supplementary Fig. S3D–S3F). This indicates the selective immunomodulation of CD8^+^ T-cell compartment, making LME-sEV influence cell-selective rather than systemic. In our experimental setup, CD8^+^ T cells appear the principal targets of LME-sEVs *in vivo*.

### LME-sEVs Affect the CD8^+^ T-cell Transcriptome, Proteome, and Metabolome

**Figure 4.**
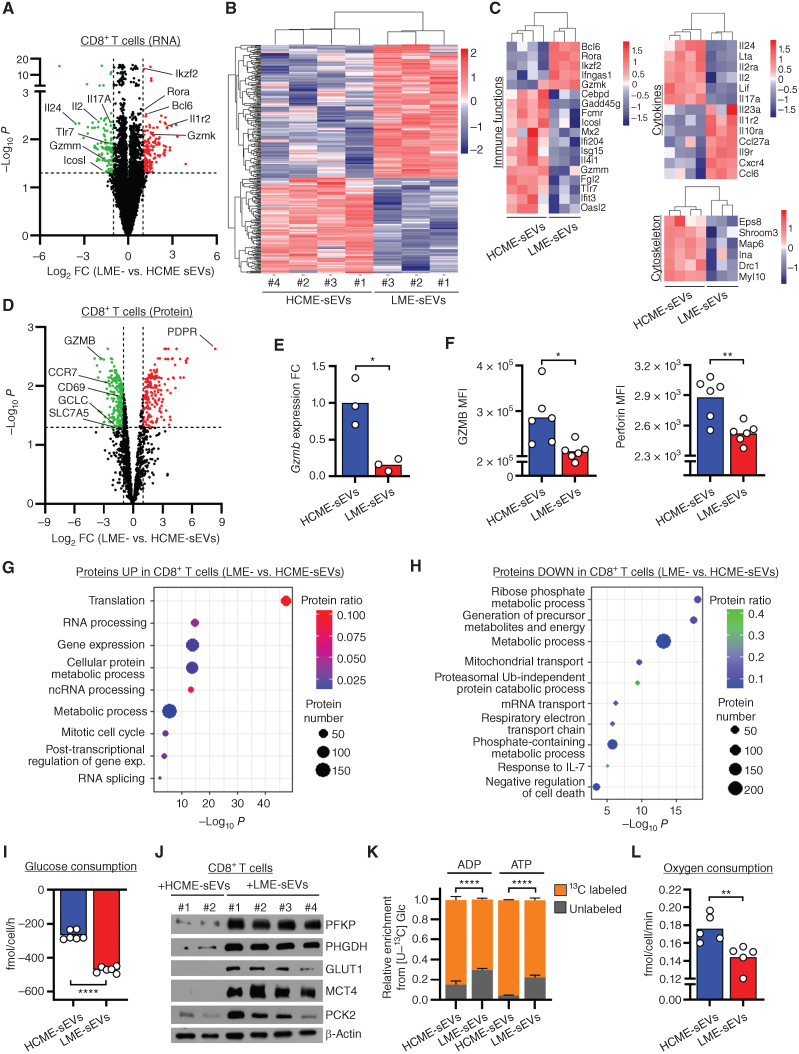
LME-sEVs impact CD8^+^ T-cell transcriptome, proteome, and metabolome. **A,** Volcano plot showing DEG identified by RNA-seq from CD8^+^ T cells treated for 48 hours with LME- (*n* = 4) or HCME-sEVs (*n* = 3) with FDR <0.05 and log_2_FC >1. **B** and **C,** Hierarchical clustering of all DEG (**B**) and of selected genes from relevant cell functions (**C**). **D,** Volcano plot showing differentially expressed proteins (DEP) identified by mass spectrometry from CD8^+^ T cells treated for 96 hours with LME- (*n* = 3) and HCME-sEVs (*n* = 3) with FDR <0.05 and log_2_FC >1. **E** and **F,** GzmB mRNA expression and GzmB and perforin levels in CD8^+^ T cells treated for 48 hours with HCME- or LME-sEVs. **G** and **H,** Ontology analysis of enriched (**G**) or diminished (**H**) DEP in CD8^+^ T cells treated with LME- or HCME-sEVs (from **D**). **I,** Levels of glucose measured by mass spectrometry in culture medium in CD8^+^ T-cell treated with LME- or HCME-sEVs for 96 hours. Negative value represents consumption. **J,** Immunoblot analysis of glycolysis-related proteins from CD8^+^ T cells treated for 96 hours with LME- or HCME-sEVs. **K,** Levels of ADP and ATP generated from ^13^C-glucose measured by mass spectrometry in CD8^+^ T cells treated with LME- (*n* = 5) or HCME-sEVs (*n* = 6) for 96 hours. **L,** Oxygen consumption measured by SeaHorse assay from CD8^+^ T cells treated with LME- or HCME-sEVs for 96h. *, *P* < 0.05; **, *P* < 0.01; ****, *P* < 0.0001 (unpaired Student *t* test). Data are mean and SEM.

As LME-sEVs alter preferentially CD8^+^ T cells *in vivo*, we focused on these essential antitumor cytotoxic cells. We profiled gene expression and protein content of sorted CD8^+^ T lymphocytes treated *ex vivo* with ME-sEVs. Only a slight modulation of genes and proteins was observed after 24 hours (77 genes and 43 proteins; Supplementary Fig. S4A–S4C; Supplementary Tables S1 and S2), all of them involved in lymphocyte activation and immune response (*Itgb1*, *Card6, Tnfrsf4/Ox40*). After 48 hours, striking changes were observed (331 genes modulated) with the repression of genes involved with CD8^+^ T-cell activation (*Tlr7* and *Icosl*), survival and proliferation (*Il2* and *Il2ra*), and immune activity (*Il17a, Lta, Gzmm*, and *Map6*: Fig. 4A–C; Supplementary Table S2). In contrast, genes associated with decreased activation and functionality (*Gzmk*), proliferation and survival (*Rora, Il10ra*), and the generation of memory CD8^+^ T cells (*Bcl6*) were increased in these cells. The differential transcription factor usage following treatment with LME-sEVs suggested an increased differentiation to effector-memory T (T_em_) cells (Zfp281), hyporesponsiveness (Stat1/3 and Myc), and a reduced T-cell activation (Snai1/Zeb and Tbr1; Supplementary Fig. S4D).

In addition, proteomic analysis of LME-sEV–treated CD8^+^ T cells showed time-dependent changes in key proteins involved in immune response and metabolism (96 hours, 422 proteins modulated, Fig. 4D; Supplementary Table S1). Downregulated proteins included the activation marker CD69, and the serine protease granzyme B (GzmB), both crucial for CD8^+^ T-cell effector functions. Repression of both cytolytic effector molecules GzmB and perforin was also independently confirmed (Fig. 4E and F). Analysis of proteomics data with a miRNA-target prediction algorithm indicated that a significant fraction of proteins repressed by LME-sEVs are putative targets of at least a specific miRNA found enriched in LME-sEVs (Fig. 2J; Supplementary Fig. S4E). This suggests an active protein downregulation mediated by miRNA transferred by LME-sEVs into CD8^+^ T cells.

Functionally, proteins increased were involved in gene expression, RNA processing, and translation, whereas proteins repressed were linked to metabolism (Fig. 4G and H). Interestingly, the amino acid transporter Slc1a5 and the glutamate cysteine ligase (Gclc) were affected, the latter being essential for fueling CD8^+^ lymphocytes after activation (28). Conversely, we observed accumulation of the inhibitory pyruvate dehydrogenase phosphatase regulatory subunit (Fig. 4D). To validate these observations, we performed metabolic profiling on ME-sEV–treated CD8^+^ T cells. By analyzing the uptake/release rates from the cell culture medium, an increased consumption of glucose and glutamine, as well as increased lactate release rate, suggested a rewiring of central carbon metabolism (Fig. 4I; Supplementary Fig. S4F). Enhanced glycolysis was also highlighted by the increase of glycolysis-related proteins, including glucose transporter 1 (GLUT1) and D-3-phosphoglycerate dehydrogenase (PHGDH; Fig. 4J). However, isotopologue tracing demonstrated that the contribution of glucose to nucleotide *de novo* synthesis, via the pentose phosphate pathway, was considerably reduced (Fig. 4K; Supplementary S4G), indicating a decreased proliferative potential. Based on the results derived from ^13^C-Glucose tracing, we hypothesized that an increased consumption of glutamine would lead to an increased usage in the tricarboxylic acid (TCA) cycle. Interestingly, the relative flux of glutamine toward TCA metabolites, proline, and glutathione (GSH) was not different after LME-sEV treatment (Supplementary Fig. S4H). Throughout the TCA cycle, molecules of NADH (nicotinamide adenine dinucleotide (NAD) + hydrogen (H)) are generated. Oxygen is particularly important to oxidize NADH to NAD^+^ used to generate ATP and perform multiple biological processes. However, using SeaHorse analysis, we observed a decreased oxygen consumption rate (OCR) in the presence of LME-sEVs (Fig. 4L). These data demonstrated that CD8^+^ T cells have decreased oxidative phosphorylation potential when treated with LME-sEVs. Finally, the use of glutamine for protein synthesis is in accordance with our observation that translation is highly increased in CD8^+^ T cells treated with LME-sEVs (Fig. 4H). Glutamine is also used for purine and pyrimidine bases synthesis. Indeed, higher levels of metabolites, including ADP and ATP, were found in CD8^+^ T cells treated with LME-sEVs (Supplementary Fig. S4I). Altogether, our data suggest LME-sEV–mediated profound perturbations in CD8^+^ T cells, leading to metabolic blockade (Supplementary Fig. S4J). Accordingly, increased glycolysis was recently linked with CD8^+^ T-cell exhaustion (29).

Finally, CD8^+^ T-cell functions are tightly regulated by other immune cells, in particular other lymphocyte subsets. Despite the injection of LME-sEVs *in vivo* did not show a considerable effect on other lymphocytes than CD8^+^ T cells (Supplementary Fig. S3D–S3F), we cannot rule out that a direct exposure to sEVs in the LME could lead to a substantial impact on these cells. Thus, we exposed Tregs, CD4^+^ T_conv_ cells, and CD19^+^ B cells to purified ME-sEVs *ex vivo* and inspected gene expression. We identified 378 genes regulated in Treg pointing to an activated phenotype (ref. 30; TNFRSF9^+^; Supplementary Fig. S5A–C; Supplementary Table S2). Effector molecules (*GzmB* and *GzmK*), signaling molecules (*Stat1, Irf4*, and *Irak3*), and activation markers (*Pdcd1*, *Cd27*, and *Tigit*) were increased. We also confirmed an increase of GzmB at the protein level (Supplementary Fig. S5D). Interestingly, the majority of differential expression of genes (DEG) were repressed by LME-sEVs in CD4^+^ T_conv_ cells (211 out of 227, *Cd27, Il7r, Il17ra, Tcf7*, and *Tox*), indicating a strong suppression of T-cell activation and proliferation (Supplementary Fig. S5E–S5G; Supplementary Table S2). CD19^+^ B cells were the least affected (77 DEGs and no enriched ontology; Supplementary Fig. S5H and S5I; Supplementary Table S2).

Altogether, these data indicate that CD8^+^ T cells are major targets of LME-sEVs *in vivo* and *ex vivo* as transcriptomic, proteomic, and metabolic evaluations pointed to profound perturbations of cell activation, proliferation, and immune functions. Worth of notice, *ex vivo* LME-sEV–treated Tregs showed a highly suppressive phenotype that could contribute to CD8^+^ T-cell repression in the tumor microenvironment.

### LME-sEVs Decrease CD8^+^ T-cell Functions

**Figure 5.**
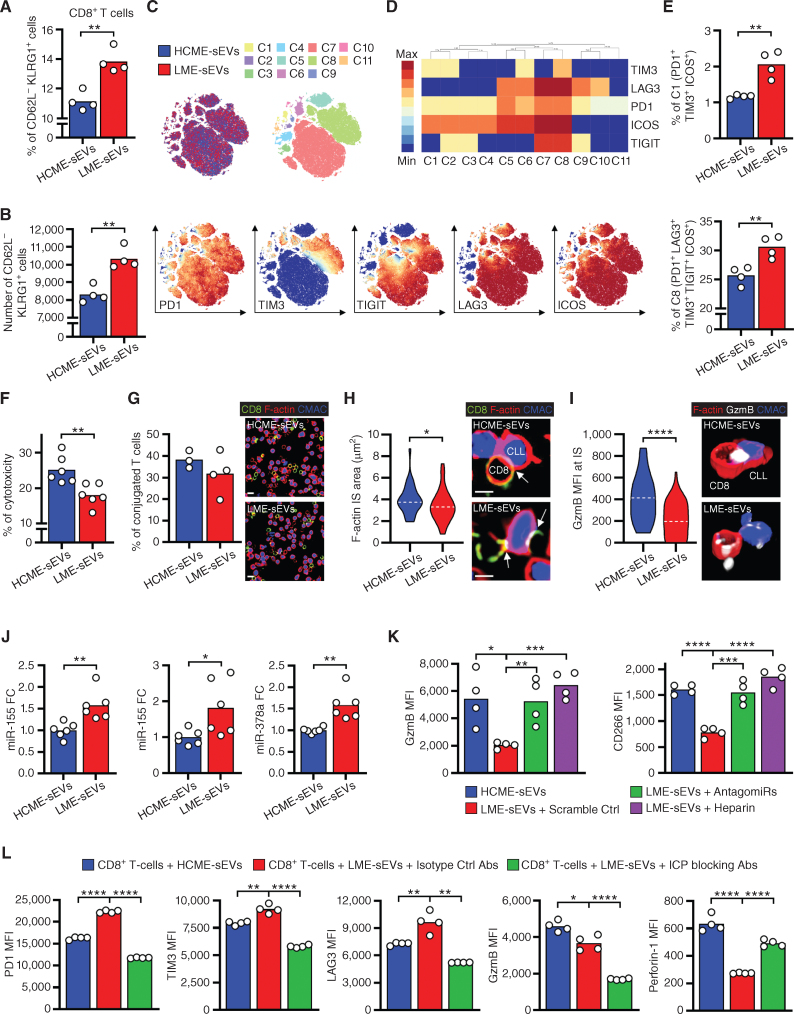
LME-sEVs decrease CD8^+^ T-cell functions. **A** and **B,** Percentages (**A**) and numbers (**B**) of CD62L^−^KLRG1^+^ CD8^+^ T cells after 48 hours of treatment with LME- and HCME-sEVs assessed by FC. **C,** Expression of ICP on CD62L^−^KLRG1^+^ CD8^+^ T cells from **B**. HSNE clustering depicting treatments, cluster identity, and marker expression. **D,** Hierarchical clustering based on ICP expression. **E,** Percentages of PD1^+^TIM3^+^ICOS^+^ CD8^+^ T cells from cluster C1 (top) and of PD1^+^LAG3^+^TIM3^+^TIGIT^+^ICOS^+^ CD8^+^ T cells from cluster C8 (bottom). **F–I,** CD8^+^ T cells were isolated from C57BL/6 and CLL cells from TCL1 mice. **F,** Percentage of T cell–mediated killing of TCL1 cells (cytotoxic assay) in the presence of HCME-sEVs or LME-sEVs (*N* = 6). **G,** Quantification of CD8^+^ T-cell:TCL1 cell conjugates upon treatment with LME- or HCME-sEVs (*N* = 3–4) and representative images (scale bar, 10 μm). **H,** Quantification of immune synapse formation (F-actin area in μm^2^, HCME-, *n* = 31 and LME-sEVs, *n* = 39, dashed line representing median) and representative medial optical sections (scale bar, 5 μm) with arrows indicating the synapse. **I,** Mean Fluorescence intensity (MFI) of GzmB at the synapse between CD8^+^ T and CLL cells (HCME-, *n* = 31 and LME-sEVs, *n* = 51) and representative 3D volume-rendered images. **J,** miRNA levels quantified by RT-qPCR in CD8^+^ T cells treated with HCME- or LME-sEVs for 24 hours. **K,** Protein levels of miRNA targets determined by FC in CD8^+^ T cells treated for 48 hours with HCME-sEVs or LME-sEVs transfected with scramble or antagomiRs (miR-150, -155, and -378a). Preincubation of LME-sEVs with heparin was used as an inhibitor of sEV internalization. **L,** ICP levels determined by FC in CD8^+^ T cells treated for 48 hours with HCME-sEVs or LME-sEVs preincubated with blocking Abs (PD-L1, GAL9, VISTA, and MHC-II) or corresponding isotypes. *, *P* < 0.05; **, *P* < 0.01; ***, *P* < 0.001; ****, *P* < 0.0001 (unpaired Student *t* test). Data are mean.

First, we screened splenic CD3^+^ versus CD3^−^ cells from leukemic mice for the expression of ICP receptors and found an enrichment of a variety of ICP receptors in CD3^+^ T cells (Supplementary Fig. S6A), confirming the highly exhausted and immunosuppressive microenvironment we previously reported in TCL1 mice (10). Next, we evaluated the phenotype and activity of CD8^+^ T cells treated with LME-sEVs. First, LME-sEVs stimulated the generation of CD8^+^ lymphocyte subpopulations, as visible by the expansion of T_em_ cells (Fig. 5A and B). We performed a clustering of CD8^+^ T cells based on ICP surface expression, using the HSNE algorithm. Eleven clusters were identified based on ICOS, LAG3, PD1, TIGIT, and TIM3 expression among T_em_ cells (Fig. 5C and D). Clusters C1 and C8 coexpressing the 5 ICP were significantly enriched (Fig. 5E), suggesting an exhausted phenotype. Concerning sEV immuno-modulatory effects, IL2 and IFNγ synthesis was particularly decreased by LME-sEVs, demonstrating their impact on CD8^+^ lymphocyte cytokine polyfunctionality (Supplementary Fig. S6B–S6D). In addition, adenosine, converted by CD39/CD73 starting from ATP, significantly reduces CD8^+^ T-cell proliferative capacity (31). Indeed, the proliferation of CD8^+^ T cells is markedly reduced by LME-sEV (expressing CD39; Supplementary Fig. S2C) treatment in the presence of ATP (Supplementary Fig. S6E). This, together with the decrease in perforin and GzmB (Fig. 4F), strongly suggests a robust LME-sEV–mediated deregulation of CD8^+^ T-cell signaling and production of cytokines and cytotoxic molecules.

Therefore, we aimed to confirm the functional impact of LME-sEVs on CD8^+^ T cells using an *ex vivo* quantitative cytotoxicity assay against TCL1 CLL cells. LME-sEV treatment led to reduced cytotoxic activity of CD8^+^ T cells against CLL cells (Fig. 5F), without altering the ability of CD8^+^ lymphocytes to interact with target cells (Fig. 5G). Importantly, LME-sEVs decreased the ability of CD8^+^ T cells to form F actin–enriched immune synapses, a key signaling structure required to drive the secretion of cytolytic granules to lytic synapses (Fig. 5H). Consistent with decreased RNA and protein levels (Fig. 4E and F), the level of GzmB expression at the synapse was markedly reduced in CD8^+^ T cells treated with LME-sEVs (Fig. 5I). In accordance with the enrichment of LME-sEVs in several miRNAs (Fig. 2J), we observed a significant increase in miRNA levels in CD8^+^ T cells treated with LME-sEVs (Fig. 5J; Supplementary Fig. S6F). Interestingly, these miRNAs (miR-150, -155, and -378a) were previously described as negative regulators of GmzB in CD8^+^ T and NK cells (32–35), two markers that we found recurrently downregulated in CD8^+^ lymphocytes treated with LME-sEVs (Fig. 4D–F; Supplementary Fig. S5I).

To identify particular cargoes responsible for the effects observed on CD8^+^ T cells, we focused on these miRNAs as they are transferred to target cells as well as on ICP as highly important regulators of T-cell functions.

Treated CD8^+^ T cells with LME-sEVs previously transfected with antagomiRs targeting miR-150, -155, and -378a, showed a rescue in GzmB and CD226 protein levels, comparable to CD8^+^ T cells treated with HCME-sEVs or LME-sEVs precoated with heparin (Fig. 5K), whereas Prf1 and Itpkc levels remained unaffected (Supplementary Fig. S6G). In addition, the transfection of LME-sEVs with single antagomiRs did not restore the levels of target genes in treated CD8^+^ T cells (Supplementary Fig. S6H), demonstrating that multiple miRNAs are needed to target these molecules. Interestingly, treatment with HCME-sEVs transfected with miRNA mimics for miR-150, -155, and -378a had a similar effect on GzmB level than LME-sEVs, confirming that the repression of GzmB we observed was due to these miRNAs (Supplementary Fig. S6I).

Next, we focused on ICP ligands present on LME-sEV surface and used blocking antibodies to neutralize their immunosuppressive functions on CD8^+^ T cells. Although LME-sEVs (+ isotype Ab) stimulated ICP expression and decreased GzmB and perforin levels as we previously showed, preincubation with anti–PD-L1, GAL9, VISTA, and MHC-II blocking Ab decreased ICP expression, restored perforin level, but had no effect on the GzmB level in treated CD8^+^ T cells (Fig. 5L), suggesting that different molecules present in LME-sEVs affect multiple pathways in target cells leading to their overall effect.

Altogether, through multiple molecules, LME-sEVs decreased CD8^+^ T-cell functional potential, impacting cytokine production and engaging ICP receptors, thus decreasing cytotoxic effect toward tumor cells.

### sEVs Are Crucial for CLL Development by Impairing the Antitumor Immune Response *In Vivo*

**Figure 6.**
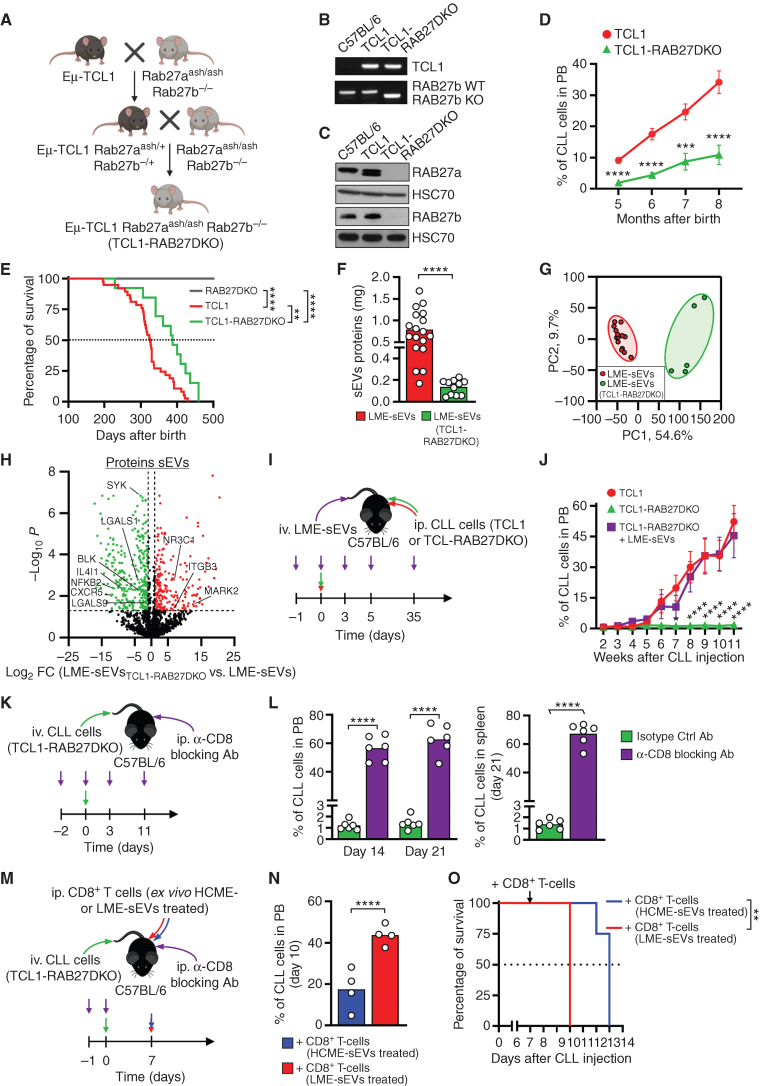
sEVs are crucial for CLL development by impairing the antitumor immune response *in vivo.***A,** Generation of a new TCL1-RAB27DKO mouse model. **B,** Detection of the human *TCL1* transgene and Rab27b excision in gDNA of C57BL/6, TCL1, and TCL1-RAB27DKO mice. **C,** Immunoblot analysis of RAB27A and RAB27B proteins in the same mice. **D,** Percentage of CD5^+^CD19^+^ CLL cells in the PB of TCL1 (*n* = 35) or TCL1-RAB27DKO (*n* = 12) mice over time. **E,** Survival of mice from **D** and RAB27DKO mice (*n* = 10). **F,** Quantity of proteins recovered from LME-sEVs (*n* = 18) or LME-sEVs_TCL1-RAB27DKO_ (*n* = 11) normalized per gram of spleen. **G,** PCA based on differentially expressed proteins (DEP) between LME-sEVs_TCL1-RAB27DKO_ and LME-sEVs with FDR <0.05 and log_2_FC >1. **H,** Volcano plot showing DEP. **I,** Injection scheme of CLL cells competent (TCL1, red arrows) or deficient in sEV release (TCL1-RAB27DKO, green arrows) into C57BL/6 mice, with or without LME-sEVs (violet arrows). **J,** Percentage of CD5^+^CD19^+^ CLL cells in the blood of C57BL/6 mice injected according to **I** (*n* = 16 per condition). Four different clones for each genotype were injected into 4 mice each. **K,** Injection scheme of CLL cells deficient in sEV release (TCL1-RAB27DKO, green arrows) into C57BL/6 mice, treated with α-CD8 blocking or isotype-control Abs (violet arrows). **L,** Percentage of CD5^+^CD19^+^ CLL cells in the PB of mice injected according to **K** (*n* = 6 per group) at days 14 and 21 (left) and in the spleen of the same mice at day 21. **M,** Injection scheme of CLL cells deficient in sEV release (TCL1-RAB27DKO, green arrows) into C57BL/6 mice, together with α-CD8 blocking Ab (violet arrows) and followed by injection of activated CD8^+^ T cells treated *ex vivo* with HCME- (blue arrows) or LME-sEVs (red arrows). **N,** Percentage of CD5^+^CD19^+^ CLL cells at day 10 in the PB of mice injected according to panel **M** (*n* = 4 per group). **O,** Survival of mice from **M** (*n* = 4 per group). *, *P* < 0.05; **, *P* < 0.01; ***, *P* < 0.001; ****, *P* < 0.0001 (unpaired Student *t* test for **F**, **L**, and **N** two-way ANOVA followed by the Bonferroni multiple comparison test for **D** and **J**, log-rank test for **E** and **O**). Data are mean with SEM.

Considering the enrichment of sEVs in the LME, their specific cargo and surface molecules, and impact on CD8^+^ T cells, we investigated their role during CLL development *in vivo*, by genetically impairing sEV release in a novel preclinical CLL murine model, TCL1-RAB27DKO (Fig. 6A). RAB27A and B are two proteins essential for sEV release, being majorly involved in the docking of vesicles at the cellular membrane (36–38). Validation of the model showed the presence of the human *TCL1* transgene together with the *Rab27b* genomic deletion, and lack of both RAB27A and B proteins (Fig. 6B and C). TCL1-RAB27DKO showed a striking delay in CLL progression, noticeable by the slower accumulation of CD5^+^CD19^+^ CLL cells in the PB, and consequently increased mouse survival (Fig. 6D and E), further confirmed with histologic analyses of the spleens (Supplementary Fig. S7A). TCL1-RAB27DKO ultimately developed the disease and required euthanasia. Despite TCL1-RAB27DKO spleens being of comparable size as TCL1, the quantity of LME-sEVs was dramatically decreased (Fig. 6F; Supplementary Fig. S7B). In addition, LME-sEV_TCL1-RAB27DKO_ protein content is largely different (546 proteins modulated; Fig. 6G–H; Supplementary Fig. S7C; Supplementary Table S1). Indeed, LME-sEVs_TCL1-RAB27DKO_ contained a lower quantity of several proteins involved in the suppression and control of lymphocyte activation, signaling, and proliferation (LGASL1, LGASL9, CXCR5, IL4I1, BLK, and SYK), whereas proteins enriched were involved in gene expression, RNA processing, and translation (Fig. 6H; Supplementary Fig. S7D and S7E). Based on the differentially present proteins, sEV preparations clustered in principal component analysis (PCA) according to mouse genotypes (Supplementary Fig. S7F).

Next, we compared gene expression from TCL1-RAB27DKO and TCL1 leukemic cells (Supplementary Fig. S7G–S7H; Supplementary Table S2). GSEA confirmed the decrease, at the mRNA level, of protein secretion in TCL1-RAB27DKO cells (Supplementary Fig. S7I). Rab27 inactivation led to decreased expression of genes involved in vesicle trafficking (*Bet1, Lamp2, Rab7, Scamp1*, and *Vamp1*), whereas *Snx31* responsible for vesicles formation in the MVB was upregulated (Supplementary Fig. S7G). Interestingly, an enrichment in gene sets driven by NF-κB, Myc, and Wnt/β-catenin was observed, suggesting the induction of oncogenic programs as a compensatory mechanism (Supplementary Fig. S7I).

To confirm that the delay in CLL development was due to CLL cell inability to release sEVs, rather than to a general lack of sEVs in the LME, we transferred CLL cells isolated from spleens of TCL1 or TCL1-RAB27DKO mice into WT C57BL/6 recipient mice. Contrary to TCL1 cells, TCL1-RAB27DKO cells failed to recapitulate CLL development (Fig. 6I and J; Supplementary Fig. S8A). Importantly, injection of LME-sEVs rescued leukemia development to levels comparable to TCL1 transfer, demonstrating the impact of LME-sEVs on CLL development (Fig. 6I–J; Supplementary Fig. S8A). To evaluate a potential autocrine effect, we treated *ex vivo* TCL1-RAB27DKO leukemic cells with LME-sEVs or LME-sEVs_TCL1-RAB27DKO_ (Supplementary Fig. S8B–S8D; Supplementary Table S2). The effect was moderate and pointed to B-cell functions (*Bach2* and *Maf*) and increased immunosuppressive capabilities (*Cx3cr1*).

Despite being unable to recapitulate the disease in immunocompetent C57BL/6 mice, TCL1-RAB27DKO CLL cells successfully induced leukemia development in immunodeficient NSG mice (Supplementary Fig. S8E–S8H), highlighting the proficiency of TCL1-RAB27DKO CLL cells to engraft when the immune system is not intact. To confirm that CD8^+^ T cells are key in the control of CLL development, we selectively depleted CD8^+^ T cells in C57BL/6 mice before injecting TCL1-RAB27DKO CLL cells (Fig. 6K; Supplementary Fig. S8I). We observed a rapid increase of percentage and number of leukemic cells in the blood and spleen of CD8^+^-depleted mice (Fig. 6L; Supplementary Fig. S8J and S8K), endorsing the role of CD8^+^ T cell–mediated immune surveillance of TCL1-RAB27DKO CLL cells. In a final experiment, we sought to evaluate *in vivo* the ability of ME-sEVs to affect CD8^+^ T cells and therefore to influence disease outcome (Fig. 6M). Mice depleted in endogenous CD8^+^ T cells and injected with TCL1-RAB27DKO CLL cells, had significantly more CLL cells in the PB and shorter survival when injected with CD8^+^ T cells previously treated with LME-sEVs compared with HCME-sEVs (Fig. 6N and O). We also observed that LME-sEV–treated CD8^+^ T cells did neither persist nor proliferate in recipient mice, possibly explaining the different outcomes (Supplementary Fig. S8L and S8M).

Altogether, we demonstrated here that TCL1-RAB27DKO CLL cells fail to induce CLL in C57BL/6 due to their inability to release sEV and impact the microenvironment. LME-sEVs inhibit CD8^+^ T cell–mediated antitumor immunity and are sufficient to restore the leukemic potential of TCL1-RAB27DKO CLL cells.

### Expression of sEV-Related Genes Correlates with Disease Progression and Poor Survival in CLL Patients

**Figure 7.**
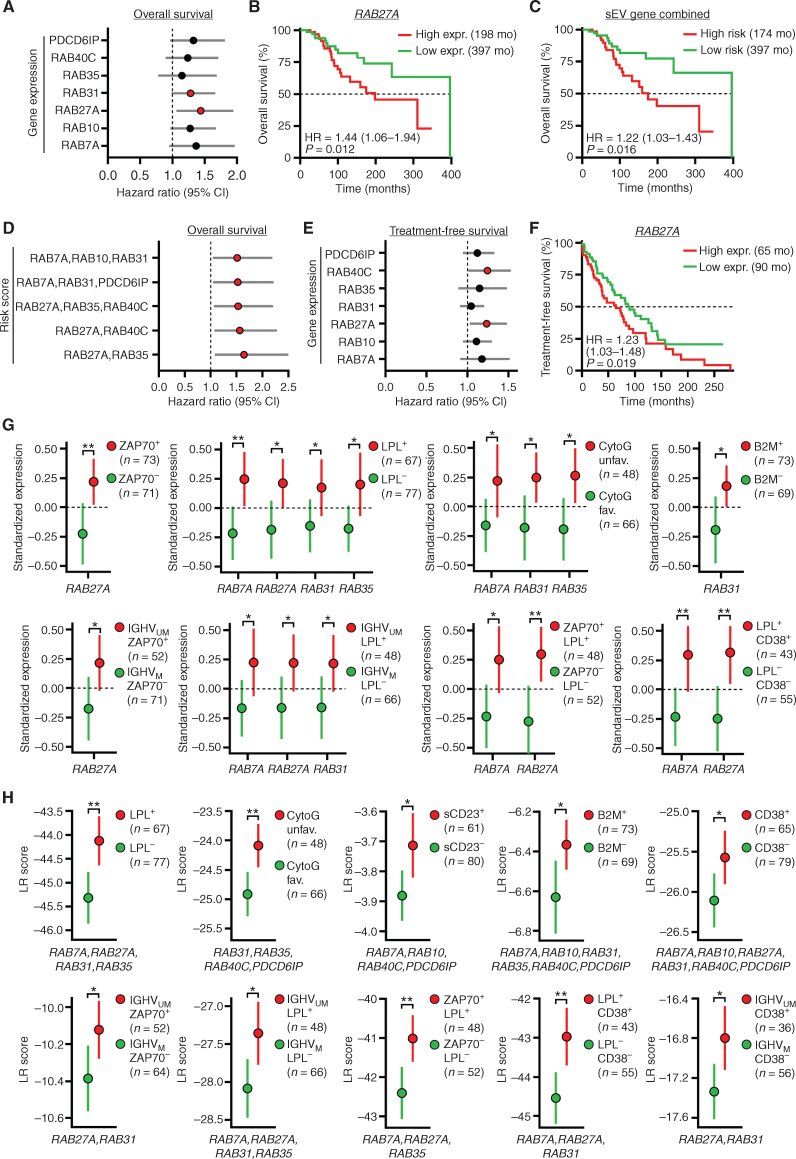
Expression of sEV-related genes correlates with disease progression and poor survival in CLL patients. Gene-expression analysis was performed by RT-qPCR for 7 genes involved in sEV biogenesis and secretion in a cohort of 144 CLL patients. The correlation between gene expression and survival was evaluated by Cox univariate regression analysis. Gene expression in clinical groups was evaluated by differential expression analysis for single genes or by logistic regression (LR) analysis for multiple genes. **A,** Calculated hazard ratios >1 (red dots, *P* < 0.05) indicate an increased risk for patients with high single-gene expression in terms of OS. **B,** Correlation between high or low gene expression and OS. Low and high groups are of identical size (*n* = 72) Median OS is indicated in months (mo). **C,** Correlation between high or low combined 7-gene expression and OS. **D,** Calculated hazard ratios >1 (red dots, *P* < 0.05) indicate an increased risk for patients with high multiple gene expression in terms of OS**. E,** Calculated hazard ratios >1 (red dots, *P* < 0.05) indicate an increased risk for patients with high single-gene expression in terms of TFS. **F,** Correlation between high or low gene expression and TFS. **G** and **H,** Standardized expression of single genes (**G**) or LR scores for multiple genes (**H**) in groups of patients according to prognostic markers (CytoG, cytogenetics, group size indicated in each panel). *, *P* < 0.05; **, *P* < 0.01. Data are mean with 95% confidence intervals.

To validate the importance of sEVs in CLL patients, we quantified the expression of *RAB7a, RAB10, RAB27a, RAB31, RAB35, RAB40C*, and *PDCD6lP*, involved in vesicle biogenesis and secretion by RT-qPCR in a cohort of 144 CLL patients. We identified *RAB27a* and *RAB31* as predictors of overall survival (OS), as higher expression correlated with poor OS (Fig. 7A and B; Supplementary Fig. S9A). Similarly, when combining *RAB27a* and *RAB31* with other sEV-related genes, the signature correlated with OS (Fig. 7C) and a higher hazard ratio (HR; Fig. 7D). Similar results were obtained for treatment-free survival (TFS; Fig. 7E and F; Supplementary Fig. S9B–S9D). Single-gene analysis confirmed the differential expression of *RAB7a, RAB27a, RAB31*, and *RAB35* between CLL groups (e.g., ZAP70^+^ vs. ZAP70^−^) and subgroups (e.g., IGHV_M_ LPL^+^ vs. IGHV_UM_ LPL^−^; Fig. 7G; Supplementary Fig. S9E) characterized by the expression of markers classifiers of prognosis. Similarly, we identified signatures differentially expressed between clinical groups (Fig. 7H). Altogether, our data confirmed the relevance of sEVs in CLL pathology.

## DISCUSSION

Recent studies focused on understanding how the microenvironment sustains tumor growth and protects cancer cells. In this regard, sEVs are important for cell-to-cell communication, and thus represent possible targets for antitumor therapies. The complexity behind sEV-based communication during cancer development relies on their ability to alter the microenvironment cellular composition and functions. Previous works by us and others partially elucidated sEV involvement in CLL progression and remodeling of the TME (5, 13–15). Interestingly, we found here a sEV signature linked with the biogenesis and release of sEVs by CLL cells in humans and mice. However, the relevance of such studies using cell line– or plasma-derived sEVs and *in vitro* cultures points to the need of more physiologic conditions to study sEV (24). This confirmed the relevance of designing a robust protocol to isolate sEVs from the murine LME. We focused our attention on the functional effect of LME-sEVs on lymphocyte subsets as they internalized LME-sEVs, highlighting the possibility of cargo-mediated effects in addition to surface molecule interactions. T lymphocytes are known to poorly internalize sEV (26, 27). This discrepancy suggests the presence of surface molecules on sEVs produced in a whole organism that could lack when produced *in vitro*. Importantly, we noted that exclusively CD8^+^ T cells were affected after seven days of treatment *in vivo*. This unforeseen observation indicated a certain specificity of sEV rather than a broad effect, as LME-sEVs could enter multiple lymphocyte subsets *in vitro*. Again, this highlights the need for a whole microenvironment to fully understand the role of sEVs in cancer (24).

We characterized LME-sEVs to decipher their impact on the TME. Surface molecules screening highlighted the enrichment in CD20 on LME-sEVs, confirming the B-cell origin of the vesicles and our previous observation (14). PD-L1, CTLA-4, and TIM-3 were already reported as crucial effectors of sEV function on immune cells (39). Given the complexity of sEV content, we believe that sEVs convey molecules of multiple natures (ICP, miRNA, enzymes) having complementary functions. First, we found multiple ICP ligands on LME-sEVs, and the corresponding receptors on matched spleens CD3^+^ T cells. This supports our previous report of multiple ICP receptors on T cells in murine CLL (10), the possible engagement of ligands carried by sEV inducing T-cell exhaustion and immune escape (7). More­over, the presence of multiple miRNAs enriched in CLL-sEVs (15, 25, 40, 41), transferred to CD8^+^ T cells, and known to disrupt effector cell cytotoxic properties (32–35) pointed to GzmB and perforin, consistently inhibited by LME-sEVs in CD8^+^ T cells. miRNA neutralization and ICP blockade both partially reversed the effects observed on CD8^+^ T cells. As a functional readout, we also focused on immune synapse formation between CD8^+^ T cells and TCL1 leukemic B cells and confirmed reduced expression of GzmB at lytic synapses in CD8^+^ T cells previously exposed to LME-sEVs *in vitro*, ultimately leading to decreased functional cytotoxicity. Interestingly, LME-sEVs had an opposite effect on Tregs, stimulating the expression of GzmB, and thus their immunosuppressive functions (42). We confirmed that treatment of CD8^+^ T cells with ATP and CD39^+^LME-sEVs decreased proliferation, cytotoxicity, and IL2/GzmB production (31, 43). Overall, the combinatory activity of ICP, miRNAs, and hydrolytic enzymes leads to a decrease in T-cell functionalities, ultimately suppressing the antitumor immune response. In accordance with this, gene-expression profiling from *in vitro* and *in vivo* LME-sEV–treated CD8^+^ T cells further showed a consistent impact on immune functions, cytokine release, cytoskeleton organization, and metabolic changes. Metabolic adaptation and manipulation by the tumor is recognized as a hallmark of cancer. Recently, tumor-derived EVs were shown to reprogram the metabolism of macrophages, thereby preparing an immunosuppressive niche characterized by increased glycolysis and lactate release (44). These metabolic adaptations—increased glucose consumption and lactate release—are known markers of exhaustion (29) that we also observed in LME-sEV–treated CD8^+^ T cells. The TCA cycle produces energy to feed the oxidative phosphorylation (OXPHOS). Recently, a high OXPHOS in CD8^+^ T cells was described to be deleterious for immunotherapy in melanoma (45). In addition, we identified inside LME-sEV proteins involved in metabolism, including IL4I1, a metabolic ICP recently described in CLL (46), also pointing to the possible delivery of the enzyme to immune cells, thus influencing their activation status. Finally, although no striking effect of LME-sEVs on Tregs was observed *in vivo*, the vesicles rapidly modified Tregs *in vitro* toward a more immunosuppressive phenotype similar to the one observed in TCL1 mice. Their activation and GzmB increase *in vitro* confirmed their potential to respond to LME-sEVs and suggested their involvement in the long-term hindered T-cell response *in vivo*.

To confirm the crucial role of sEVs *in vivo*, we generated a TCL1-RAB27DKO model in which sEVs release is inhibited (47). Although Rab27DKO mice are partially immunodeficient (48), secretion of cytokines by Rab27DKO immune cells remains unaffected (49). TCL1-RAB27DKO CLL cells showed strong NFκB- and Myc-driven oncogenic transcriptional programs and grew in immunodeficient mice. However, we noted a striking delay of disease onset in mice deprived of sEVs, and TCL1-RAB27DKO CLL cells transfer into immunocompetent mice failed to recapitulate the disease. Importantly, the reintroduction of LME-sEVs rescued leukemia development. We also showed, by a depletion experiment, that CD8^+^ T cells are crucial for controlling CLL development in the absence of sEVs. Finally, we confirmed that LME-sEVs are impairing T-cell functions as leukemic mice adoptively transferred with CD8^+^ T cells activated in the presence of LME-sEVs had a shorter survival than with HCME-sEVs. Altogether, despite sEV-deficient CLL cells were fit to grow *in vivo*, sEVs are indispensable to escape the antitumor immune response in immunocompetent animals.

Finally, using a cohort of CLL patients, we found a correlation between sEV-related gene expression, prognostic markers, and survival. Enhanced expression of the *RAB27A* gene by breast cancer cells promotes invasiveness and metastasis potential (50), suggesting a crucial role for sEVs in aggressive malignancies. In conclusion, we stressed on the importance to focus and characterize more complex sources of sEVs, and not limiting our evaluations to sEVs produced *in vitro*. Current therapies using ICP blockers showed a limited effect against CLL, with benefits for patients with Richter transformation (51). Having a broader overview of the TME, including sEV composition, could be key to better understand cancer progression and resistance to establish effective therapeutic strategies for patients (52).

## METHODS

### Patient Samples

All experiments involving human samples were conducted in accordance with the declaration of Helsinki, approved by the institutional review board (Jules Bordet Institute Ethics Committee), and PB samples were collected from treatment-naïve CLL patients after written informed consent. Patient cohort demographic characteristics (age and gender) and clinical parameters [Binet stage, IgHV status, zeta-associated protein 70 (ZAP70), lipoprotein lipase (LPL), CD38 molecule (CD38) expression, cytogenetic abnormalities, lymphocyte doubling time, and soluble CD23 (sCD23] are derived from our previous reports (53, 54) and reported in Supplementary Table S3. All patients had a CD19^+^CD5^+^CD23^+^ phenotype and a Catovsky score of 4/5 or 5/5. All tested prognostic factors were proven to be significant predictors of TFS and OS, indicating that our cohort is representative of a CLL population.

### Animal Experiments

All experiments involving laboratory animals were conducted in a pathogen-free animal facility with the approval of the Luxembourg Ministry for Agriculture (#LECR-2016-03, #LECR-2018-02, and #LECR-2018-03). Mice were treated in accordance with the European Union guidelines. C57BL/6 mice (MGI:3028467, RRID:IMSR_JAX:000664) were purchased from Janvier Labs (France) and NSG (MGI:3577020, RRID:IMSR_JAX:005557) mice and Foxp3^YFP/Cre^ (MGI:3790499, RRID:IMSR_JAX:016959) from Jackson Laboratories (USA). Eμ-*TCL1* mice (on C57BL/6 background; MGI:3527221) were a kind gift from Pr. Carlo Croce and Pr. John Byrd (OSU, OH) and provided by Dr. Martina Seiffert (DKFZ Heidelberg, Germany). The *Rab27a*^ash/ash^ (MGI:1856656) *Rab27b*^−/−^ (MGI:3834149; RAB27DKO) were previously described (47). Eμ-*TCL1 Rab27a*^ash/ash^*Rab27b*^−/−^ (called TCL1-RAB27DKO) mice were generated in-house as depicted in Fig. 6A, by crossing the RAB27DKO with TCL1, to introduce the TCL1 oncogene, generating the Eμ-*TCL1 Rab27a*^ash/+^*Rab27b*^−/+^. Further breeding with the RAB27DKO mice established the Eμ-*TCL1 Rab27a*^ash/ash^*Rab27b*^−/−^. CLL progression was monitored over several months in TCL1 and TCL1-RAB27DKO by determining the percentage of CD5^+^CD19^+^ CLL cells in PBMC by flow cytometry on a CytoFLEX (Beckman Coulter) using CD19-APC and CD5-PE (BioLegend). Mice reaching the humane endpoint were euthanized by cervical dislocation. All deaths unrelated to leukemia were excluded from this study. To perform the adoptive transfer (AT) in C57BL/6 and NSG control mice, CLL cells were isolated from either TCL1 or TCL1-RAB27DKO diseased spleens. Then, 10 × 10^6^ CLL cells were injected intravenously in 100 μL of DMEM without phenol red, and CLL progression was followed by weekly bleeding, as described previously. Otherwise stated, mice used for experiments were eight to ten weeks old. Both male and female mice were used (age and gender were matched within the same experiment), no variation or impact on the results due to the different sex was detected.

### Validation of the TCL1-RAB27DKO Mouse Model

Genomic DNA was extracted from a tail biopsy using Mouse Direct PCR Kit (#B40015, Biotool) following the manufacturer's instructions. The specific primer sequences were: for *Tcl1* F: 5′-GCCGAGTGCCCGACACTC-3′ and R: 5′-CATCTGGCAGCAGCTCGA-3′, for *Rab27b* F: 5′-CTGCTGCAGGATCTCACATCAGTG-3′, R_1_: 5′-AGCATCTGTAACCTAGACATTGGC-3′ and R_2_: 5′-GAAATGGGACATTGGGACAGGAGG-3′. Both amplifications were performed with the following program: 94°C for 5 minutes, 35 cycles of 94°C for 20 seconds, 59°C for 30 seconds, and 72°C for 60 seconds. After amplification, the product was run on a 1% agarose gel with SYBR Safe DNA Gel Stain (Thermo Fisher) and visualized by Image Quant Las 4000 (GE Healthcare).

Proteins from freshly isolated cells (total blood) were extracted using RIPA buffer including the cOmplete Protease Inhibitor Cocktail (Roche) and the Phosphatase Inhibitor Cocktail 2 and 3 (Sigma-Aldrich). Then, 10 μg of cell lysates were resolved on 10% SDS-PAGE and transferred to a nitrocellulose membrane. To confirm equivalent loading between lanes, a Ponceau red staining was performed. Membranes were incubated in 1× PBS-0.1%Tween and fat-free dry milk (5%, Roth) blocking buffer for 1 hour at room temperature (RT). Membranes were then incubated with primary antibodies against RAB27a (#sc-81914, RRID:AB_1128884, Santa Cruz), RAB27b (#NBP1–79631, RRID:AB_11014614, Novus), and HSC70 (#sc-7298, RRID:AB_627761, Santa Cruz) in blocking buffer at 4°C overnight. Membranes were washed three times in 1× PBS-0.1%Tween for 10 minutes each time. Secondary antibodies coupled to HRP were from Jackson ImmunoResearch. For detection, the ECL western blot detection kit was purchased from Amersham and the radiographic films from Santa Cruz Biotechnology.

### In Vivo Depletion of CD8^+^ T cells

TCL1-RAB27DKO cells (20 × 10^6^/mouse) were injected (i.v.) in C57BL/6 mice treated with 200 μg (days −2, 0, and 3) and 100 μg (weekly) of either blocking ab against CD8 (InVivoMAb anti-mouse CD8α, #BE0061, RRID:AB_1125541, Bio X Cell) or isotype control (InVivoMAb polyclonal Armenian hamster IgG, #BE0091, RRID:AB_1107773, Bio X Cell) as shown in Fig. 6K.

For the CD8^+^ T-cell transfer experiment, TCL1-RAB27DKO cells (5 × 10^6^/mouse) were injected i.v. (tail vein) in C57BL/6 mice treated with 100 μg (days −1 and 0) of anti-CD8 blocking Ab as shown in Fig. 6M. On day 7, 2 × 10^6^*ex vivo* ME-sEV–treated CD8^+^ T cells were injected i.p. in each mouse.

### ME-sEV Isolation

To isolate LME-sEVs, we used spleens from animals with over 70% CD5^+^CD19^+^ cells in PB. Dissociation was performed using GentleMACS^TM^ (Miltenyi). The protocol derives from previous publications with modifications (14, 55) and is depicted in Fig. 1I. Dissociated spleens were centrifuged for 5 minutes at 400 g, allowing to collect cells for downstream analysis and supernatant, called spleen plasma. Spleen plasma was centrifuged for 20 minutes at 400 × *g* to remove remaining cells, 40 minutes at 2,000 × *g* to remove dead cells, and 60 minutes at 10,000 × *g* to remove cellular debris and large EVs. Before ultracentrifugation (UC), the spleen plasma was filtered (0.22 μm) to remove any impurity. sEVs were isolated by UC (70 minutes, 110,000 × *g*, 4°C) followed by flotation on 17% iodixanol cushion (Optiprep, Axis-Shield, 75 minutes, 100,000 × *g*, 4°C) to remove protein complexes. Finally, sEVs were washed in PBS (70 minutes, 110,000 × *g*, 4°C). To remove aggregates, we filtered sEVs at 0.45 μm followed by 0.22 μm. To prepare fluorescent sEVs, vesicles were incubated with MemBright (now called MemGlow) 488 or 570 dyes (200 nmol/L; ref. 56) for 15 minutes at 4°C before being loaded on 17% iodixanol cushion and further processed with standard protocol. To preserve sEV integrity, each preparation was divided into aliquots and kept at −80°C until further use. Quantification of sEV-associated proteins was performed as previously described (55). Briefly, 4.5 μL of fresh isolated sEVs was lysed in 0.5 μL of 10× RIPA buffer, and 2 μL of the mix was measured via spectrophotometer (595 nm) using 1 mL of Bradford reagent (Bio-Rad). Protein concentration was determined using a BSA standard curve.

### ME-sEV Size Analysis

Tunable resisting pulse sensing (TRPS) was performed with Exoid (Izon Science) using an NP100 nanopore (100 nm) and PBS buffer as electrolyte. Data were analyzed using the provided Izon Control Suite software (RRID:SCR_021922).

### Detection of sEV Markers by Western Blot

Details for sEV western blot were previously described (55). To assess sEV purity, the presence of the following markers was assessed: Alix (i.e., PDCD6IP, #2171, RRID:AB_2299455, Cell Signaling Technology), TSG101 (#GTX70255, RRID:AB_373239, GeneTex), CD63 (#556019, RRID:AB_396297, BD Biosciences), and CD81 (#sc-7637, RRID:AB_627190, Santa Cruz Biotechnology). Furthermore, the absence of two common contaminating proteins, Calnexin (#2433, RRID:AB_2243887, Cell Signaling Technology) and PHB (#sc-377037, RRID:AB_2714190, Santa Cruz Biotechnology), was also evaluated.

### Electron Microscopy

sEV analysis was performed using formvar- and carbon-coated (ultra-thin, 200 mesh, EMS 215–412–8400) copper grids in a Cressington 208 glow-discharge unit before applying 1 μL of the sample (diluted in different concentrations in H_2_O) per grid. The grids were then washed in H_2_O three times and stained with uranylacetate for negative contrast. Imaging was taken with a Gemini SEM 300 (Zeiss) at 30 kV acceleration voltage using the sTEM detector.

### Flow Cytometry Analysis of sEVs

#### Conventional Flow Cytometry (Bead-based Strategy).

sEVs were coated on beads as previously published (57). Briefly, 4-μm aldehyde/sulfate latex beads were coated with 5 μg of ME-sEVs by incubating them overnight at 4°C. Saturation of remaining free binding sites was done using 1M glycine. Beads were then washed 4 times in PBS/0.5% BSA (3 minutes 4,000 × *g* RT). Finally, 10 μL of ME-sEV–coated beads were incubated with 50 μL of antibody diluted in PBS/0.5% BSA (30 minutes at 4°C). After two washes, sEV-coated beads were analyzed with the CytoFLEX flow cytometer (Beckman Coulter; RRID:SCR_019627). The full list of antibodies used for sEV staining can be found in Supplementary Table S4.

#### Conventional Flow Cytometry (Bead-free Single-EV Strategy).

Prior to the staining, antibodies and PBS were filtered through a 0.22-μm filter. MB488^+^ sEVs were then stained with antibodies for 30 minutes at 4°C. Acquisition was performed with a NovoCyte Quanteon Flow Cytometer (Agilent) equipped with a 0.22-μm filter for the sheath fluid to reduce electronic noise. Furthermore, the instrument was set to a minimum flow rate (5 μL/minute).

#### Imaging Flow Cytometry (Bead-free Single-EV Strategy).

MB488^+^ sEVs were stained with antibodies for 30 minutes at RT, resuspended in PBS up to 200 μL, filtered (0.22 μm), and incubated at 4°C overnight before acquisition with the ImageStreamX Mark II imaging flow cytometer (EMD Millipore). Stained MB488^+^ sEVs were acquired by setting the imaging flow cytometer as previously published (57). Briefly, the instrument was set to low-speed/high-sensitivity mode (60× magnification), and the power for the used lasers was set to the maximum.

### *In Vitro* Uptake of sEVs

Cells were seeded on glass coverslips in μ-slides (Ibidi). Then, MB488 fluorescently labeled LME-sEVs were used to treat the cells for the desired time points. Heparin (Hep) was used as an uptake inhibitor; in this case, the labeled sEVs were incubated 30 minutes at 37°C with 10 ng/mL heparin (Sigma-Aldrich) prior to treating the cells (14).

#### Flow Cytometry Analysis.

At the time of collection, cells were washed in MACS buffer (Miltenyi Biotec). Cell-surface staining was performed in 100 μL of MACS buffer (Miltenyi Biotec) for 30 minutes on ice in the dark prior analysis on the CytoFLEX (Beckman Coulter).

#### Confocal Microscopy Analysis.

At the time of collection, cells were washed in PBS, resuspended in DAPI solution (1 μg/mL) to counterstain nuclei and transferred on a new glass coverslips in μ-slides (Ibidi), letting them naturally settle at the bottom. Images were acquired on a confocal laser scanning microscope (LSM510; RRID:SCR_018062, Zeiss).

### Transfection of miRNA Inhibitors into LME-sEVs

Transfection of miRCURY LNA miRNA inhibitors against miR-150 (MMU-MIR-150-5P, #339121 YI04101206-ADA, Qiagen), -155 (MMU-MIR-155-5P, #339121 YI04101319-ADA, Qiagen), and -378a (MMU-MIR-378A-5P, #339121 YI04101421-ADA, Qiagen) or scramble control (negative control A, #339126 YI00199006-ADA, Qiagen) into LME-sEV was performed using HiPerFect Transfection Reagent (#301704, Qiagen) following a published protocol (58). Briefly, 20 pmol/L of each miRCURY LNA miRNA Inhibitors were diluted in a medium without serum, 2 μL transfection reagent was added and mixed by vortexing. The formation of molecular complexes was allowed for 10 minutes at RT. Next, the complexes were added drop-wise onto LME-sEVs and incubated at 37°C for 6 hours. sEVs were washed once in PBS before adding them to CD8^+^ T cells for 48 hours.

### *In Vivo* Injection of sEVs

#### sEVs *in Vivo* Uptake.

In order to define the uptake of sEVs *in vivo*, 100 μL of MB570^+^ LME-sEVs (1 mg/mL) were injected i.v. in 8-week-old C57BL/6 mice, which were euthanized 24 hours later. Total splenocytes were stained for different cell-surface markers (CD4, CD8, and CD19) and analyzed using NovoCyte Quanteon Flow Cytometer (Agilent).

#### *In Vivo* Treatment with sEVs.

Serial injections of LME-sEVs during the rescue experiment were performed as shown in Fig. 6I. Briefly, 100 μL of LME-sEVs (1 mg/mL) were injected i.v. the day before the experiment (day −1), together with the cells (d0), and then again on days 3, 5, and 35.

### Culture Conditions and *In Vitro* sEV Treatment

Spleens were collected from C57BL/6 or Foxp3^YFP/Cre^ mice (for Treg isolation) and rapidly transferred in a tube containing PBS (without Ca^2+^/Mg^2+^). Splenocytes isolation was performed by mechanical spleen dissociation through a 100-μm strainer (BD Biosciences), and cells were recovered by centrifugation (400 × *g*, 4°C, 10 minutes). The cell pellet was resuspended in ACK lysing solution (Lonza) to lyse red blood cells. Finally, splenocytes were washed in MACS buffer (Miltenyi Biotec), filtered through a 50-μm strainer (Celltrics, Sysmex), and counted. CD3^+^ T cells were isolated by negative selection using the MojoSort Mouse CD3 T-Cell Isolation Kit (BioLegend) following the manufacturer's instructions. The isolated T-cell population contained at least 95% of CD3^+^ T cells. CD8^+^ T cells were isolated by negative selection using the MojoSort Mouse CD8 T-Cell Isolation Kit (BioLegend) following the manufacturer's instructions. The isolated T-cell population contained at least 95% of CD8^+^ T cells. CD8^+^ T cells were cultured in anti-CD3–coated wells (10 μg/mL; #100302, RRID:AB_312667, BioLegend) in RPMI-1640 medium supplemented with 10% fetal bovine serum (FBS), 1% penicillin/streptomycin (P/S), IL2 (10 ng/mL; #589104), CD28 (3 μg/mL; #102102, RRID:AB_312867, BioLegend) and β-Mercaptoethanol (50 μmol/L). Depending on experiment duration, cells were fed with fresh medium every 48 hours. For intracellular cytokine production, cultured CD8^+^ T cells were stimulated overnight with PMA/ionomycin (100 nmol/L/1 μmol/L) and incubated for a maximum of 4 hours with Brefeldin A (BFA, 1×). CD4^+^ T cells were isolated by negative selection using the MojoSort Mouse CD4 T-Cell Isolation Kit (BioLegend) following the manufacturer's instructions. The isolated T-cell population contained at least 95% of CD4^+^ T cells (comprising CD4^+^ T_conv_ cells and Tregs). CD4^+^ T cells were cultured in anti-CD3–coated wells (10 μg/mL) with RPMI-1640 medium supplemented with 10% FBS, 1% P/S, IL2 (10 ng/mL), and β-Mercaptoethanol (50 μmol/L). For immune-checkpoint blockade, CD8^+^ T cells were treated for 48 hours using ME-sEVs previously coated with 5 μg/mL of antibodies directed against PD-L1 (#124302, RRID:AB_961228, BioLegend), MHC-II (#107601, RRID:AB_313316, BioLegend), GAL9 (#136115, RRID:AB_2860679, BioLegend), and VISTA (#BE0310, RRID:AB_2736990, Bio X Cell) for 6 hours at 4°C.

Depending on experiment duration, cells were fed with a fresh medium every 48 hours. B/CLL cells were isolated by negative selection using the MojoSort Mouse Pan B-Cell Isolation Kit II (BioLegend) following the manufacturer's instructions. The isolated B and CLL cell populations contained at least 90% of CD19^+^ or CD19^+^CD5^+^ double-positive cells, respectively.

Human PBMC were isolated by density-gradient centrifugation over Linfosep (Biomedics). B cells were purified with a CD19^+^ magnetic-bead system (MidiMACS, Miltenyi Biotech) according to the manufacturers’ instructions. Mean B-cell purity was >99% and the mean percentage of CD5^+^/CD19^+^ cells after purification was >98%, as measured by flow cytometry.

Cells used for microarray analysis were cultured for 24 hours with RPMI-1640 medium supplemented with 10% FBS, 1% P/S, and IL2 (10 ng/mL) with either LME-sEVs or HCME-sEVs.

Cells were incubated with LME-sEVs or HCME-sEVs based on the physiologic amount found in the respective spleen microenvironment (Fig. 1J). Depending on experiment duration, additional sEVs were added every 48 hours. TCL1-RAB27DKO cells were cultured for 24 hours with RPMI-1640 medium supplemented with 10% FBS, 1% P/S with either LME-sEVs_TCL1_ or LME-sEVs_TCL1-RAB27DKO_ based on the physiologic amount found in the respective spleen microenvironment (Fig. 6F).

### Flow Cytometry Sorting of Cells

Cells used for microarray analysis were sorted directly from Foxp3^YFP/Cre^ derived purified splenocytes using BD FACSAria III Cell Sorter (RRID:SCR_018934). CD4^+^Foxp3^+^ were isolated based on YFP expression, antibodies for CD8^+^ and CD4^+^Foxp3^−^ staining can be found in Supplementary Table S4.

For the experiment requiring to separate CD4^+^Foxp3^+^ from CD4^+^ T_conv_, cells were sorted directly from LME-sEV–treated Foxp3^YFP/Cre^- derived purified splenocytes using BD FACSAria III Cell Sorter (RRID:SCR_018934). Tregs were isolated based on YFP expression, and antibodies for CD8^+^, CD4^+^, and CD19^+^ staining can be found in Supplementary Table S4.

### Flow Cytometry Analysis of Cells

#### Surface Staining.

Cell-surface staining was performed in 100 μL of MACS buffer (Miltenyi Biotec) for 30 minutes on ice in the dark prior to analysis on NovoCyte Quanteon Flow Cytometer (Agilent).

PB was directly stained for 30 minutes on ice in the dark, and then red blood cells were lysed using RBC Lysis/Fixation Solution (BioLegend), according to the manufacturer's instructions. After washing twice, samples were ready for acquisition on a CytoFLEX analyzer (Beckman Coulter).

#### Intracellular Staining.

Previously surface stained cells were permeabilized using the eBioscience Foxp3/Transcription Factor Staining Buffer Set (Thermo Fisher Scientific) following the manufacturer's instructions before intracellular staining. The list of antibodies used for cell-surface and intracellular staining can be found in Supplementary Table S4.

### Flow Cytometry Clustering

Clustering analysis of live lymphocytes was performed with Cytosplore software. Briefly, 50,000 events per sample were subjected to HSNE to generate clusters based on intracellular and transmembrane markers expression. Clusters were generated using the Gaussian mean shift algorithm using the density estimate as input.

### T cell–Mediated Cytotoxicity Assay

The definition of cytotoxicity of ME-sEV–treated T cells on CLL cells was performed as previously published (59). Briefly, C57BL/6 CD3^+^ T cells were cultured in a 48-well plate on anti-CD3–coated wells (1 μg/mL) with RPMI-1640 medium supplemented with 10% FBS, 1% P/S, and 1 μg/mL anti-CD28 antibody for 48 hours at 37°C, in the presence of LME-sEVs or HCME-sEVs. Treatment with ME-sEV was repeated every 24 hours. CLL cells (CD5^+^CD19^+^) isolated from the TCL1 mouse model were stained with CellTrace CFSE (200 nmol/L, Thermo Fisher). On the day of the assay, CLL target cells were pulsed with 2 μg/mL of super antigen (sAg; SEA and SEB; Sigma-Aldrich) for 30 minutes at 37°C. Target primary CLL cells (2.5 × 10^4^) loaded with sAg were added to the ME-sEV–pretreated CD3^+^ T cells at a 1:20 (target:effector). Cell mixtures were centrifuged, and the cell pellet was incubated for 4 hours at 37°C. Cells were stained with TO-PRO-3 viability dye (Thermo Fisher) according to the manufacturer's instructions, and T cell–mediated cytotoxicity against CLL target cells was determined by flow cytometry. Cytotoxicity was calculated as: % target cell death = (% CFSE^+^ TO-PRO-3^+^ target cells incubated with effector T cells − % of CFSE^+^ TO-PRO-3^+^ target cells incubated alone) × 100/(100 − % of CFSE^+^ TO-PRO-3^+^ target cells incubated alone).

### T-cell:CLL (Tumor) Cell Conjugation and Immunologic Synapse Assays

Immune synapse assays and quantitative analysis were performed as previously described (59). Briefly, purified murine CD3^+^ T cells and purified CD5^+^CD19^+^ CLL cells from the TCL1 mouse model were incubated for 48 hours at 37°C or in the presence of LME-sEVs or HCME-sEVs. Treatment with ME-sEVs was repeated every 24 hours. Next, the same CLL cells were stained with CellTracker Blue CMAC according to the manufacturer's instructions and pulsed with 2 μg/mL of super antigen (sAg; SEA and SEB; Sigma-Aldrich) for 30 minutes at 37°C before washing. Tumor cells were then pooled with an equal number of T cells (1 × 10^6^; to ensure identical cell numbers per sample and allow accurate evaluation of changes in the percentage of conjugated T cells and F-actin immune synapse formation with treatment), centrifuged at 260 × *g* (5 minutes) and incubated at 37°C for 10 minutes (CD8^+^ T cells). Cells were then transferred onto microscope slides (Polysine slides; Thermo Scientific) using a cell concentrator (Cytofuge 2) and fixed for 15 minutes at RT with 3% formaldehyde (Thermo Scientific) in PBS.

Immunofluorescence (IF) labeling was done using Cytofuge2 cell concentrator units. After fixing, cells were permeabilized with 0.3% Triton X-100 (Sigma-Aldrich) in PBS for 5 minutes and treated for 10 minutes with 5% goat serum (Sigma-Aldrich) in PBS-blocking solution. Primary and secondary antibodies (Alexa Fluor 488 or 647, Thermo Fisher Scientific) were applied sequentially for 45 minutes at 4°C in 5% goat serum (Sigma-Aldrich) in PBS blocking solution. F-actin was stained with rhodamine phalloidin (Thermo Fisher) following the manufacturer's instructions and applied alone or with the secondary antibody. After washing, cell specimens were sealed with coverslips using mounting solution FluorSave reagent (Merck Millipore). The specificity of staining was optimized and controlled by using appropriate dilutions of isotype control, primary Abs, and subsequent fluorescent secondary Abs. Background staining using Abs alone was compared with positively stained cells and was not visible when using identical acquisition settings. Medial optical section (or Z-stacks for 3D volume images) images were captured with a high-sensitivity A1R confocal microscope (with gallium arsenide phosphide, GaAsP detector) using a 63×/1.40 oil objective with NIS-elements imaging software (Nikon). Detectors were set to detect an optimal signal below saturation limits. Fluorescence was acquired sequentially to prevent the passage of fluorescence from other channels (DU4 sequential acquisition). Image sets to be compared were acquired during the same session using identical acquisition settings.

### Real-time PCR

#### RNA Isolation.

Cellular and sEV RNA were isolated using NucleoZOL, one phase RNA purification reagent and columns from the NucleoSpin RNA Set for NucleoZOL Mini kit, according to the manufacturer's instructions (Macherey-Nagel). For transcriptomics analysis using microarrays or RNA sequencing, RNA was quantified with Qubit (Thermo Scientific), and the quality was assessed with the Fragment Analyzer 5200 using RNA kits (Agilent). For purified human CLL cells, total RNA was extracted in a single step using TriPure Isolation Reagent (Roche Applied Science).

#### MicroRNA Detection in sEV and sEV-treated CD8^±^ T Cells.

MicroRNA were quantified by RT-qPCR performed using TaqMan MicroRNA assays, TaqMan MicroRNA Reverse Transcription Kit (Thermo Scientific) and the Takyon Low ROX Probe 2X MasterMix dTTP blue (Eurogentec) (15, 20). The following probes were used: miR-21 (#002438), miR-146a (#000468), miR-378a (#000567), miR-210 (#000512), miR-27a (#000408), miR-150 (#000473), miR-155 (#002623), and U6 (#001973).

#### Gene Expression in Cells.

Reverse transcription of mRNA was performed in a SimpliAmp Thermal Cycler (Thermo Fisher) using FastGene Scriptase II cDNA 5× ReadyMix (Nippon genetics). Real-time PCR was performed in the QuantStudio 5 Real-Time PCR System (Thermo Fisher) using the SYBR Green detection system. The primers were for *GzmB* F: 5′-CAGGAGAAGACCCAGCAAGTCA-3′ and R: 5′-CTCACAGCTCTAGTCCTCTTGG-3′, *Prf1* F: 5′-TGGTGGGACTTCAGCTTTCC-3′ and R: 5′-TGCTTGCATTCTGACCGAGT-3′, *Slc7a5* F: 5′-CATCAACGACTCTGTTGTAGACC-3′ and R: 5′-CGCTGGATACAGGATTGCGG-3′, *Itpkc* F: 5′-CATCACCCCAGAGACTCCTGA-3′ and R: 5′-TTCTTCCAGGGCTTGCTTCCAG-3′ and *Fgr* F: 5′-GAGGCGGGTAGCACCTCAC-3′ and R: 5′-CCCATTCCAGATGCCCCCAC-3′ were from Eurogentec. For RNA isolated from purified human CLL cells, cDNA was generated by a retrostranscription using the qScript cDNA Synthesis Kits (Quanta Biosciences/VWR International). Real-time PCR was performed in the QuantStudio 5 Real-Time PCR System (Thermo Fisher) using the SYBR Green detection system. The primers used were *28S* F: 5′-GGGTGGTAAACTCCATCTAAGG-3′ and R: 5′-GCCCTCTTGAACTCTCTCTTC-3′, *RAB27a* F: 5′-TGGGAGACTCTGGTGTAGGG-3′ and R: 5′-ACTGGCTCTGTACACCACTC-3′, *RAB10* F: 5′-TCCCAATGGCGAAGAAGAC-3′ and R: 5′-TGATCTTGAAGTCTATTCCTATGGT-3′, *RAB35* F: 5′-GCACCATCACCTCCACGTAT-3′ and R: 5′-CCGCTTGACGTTGACAAAGG-3′, *RAB40c* F: 5′-CGTACGCCTACAGTAACGGGAT-3′ and R: 5′-GTAGGACCTGAAGATGGTGCAG-3′, *RAB31* F: 5′-TGTGCCTTCTCGGGGACAC-3′ and R: 5′-GCCCCAATAGTAGGGCTGAT-3′, *PDCD6IP* F: 5′-TCGAGACGCTCCTGAGATATT-3′ and R: 5′-AGCCAGTTTTACAGAGCCTCC-3′, *RAB7A* F: 5′-ATGCACTTAAGCAGGAAACGG-3′ and R: 5′-TGGCCCGGTCATTCTTGTC-3′.

### Transcriptomics

#### RNA Sequencing.

Librairies were prepared with the QuantSeq 3′ mRNA-Seq Library Prep Kit FWD for Illumina (Lexogen), according to the manufacturer's instructions, with the addition of UMI. Barcoded samples were pooled, diluted, and loaded onto a NextSeq 500/500 Mid Output flowcell (130M reads, Illumina), and single-end sequencing was performed on a NextSeq 550 (Illumina). After initial QCs using FastQC (https://www.bioinformatics.babraham.ac.uk/projects/fastqc/; RRID:SCR_014583) and FastQ Screen (https://www.bioinformatics.babraham.ac.uk/projects/fastq_screen/; RRID:SCR_000141), fastq files were processed using a local Snakemake workflow including the following main steps. First, raw reads were trimmed from their UMI index, poly A and adapter sequences using a combination of dedicated scripts and cutadapt (v2.10). Next, filtered reads were submitted for mapping (STAR v2.5.3a; RRID:SCR_004463) on the Mouse Reference genome (GRCm38). Collapsing of reads originating from the same fragment was achieved with umi_tools (v1.0.0) and counting was performed with featureCounts (subread v2.0.0; RRID:SCR_012919).

#### Gene-Expression Analysis.

Differential expression analysis was performed using the *limma*-based R/Bioconductor EdgeR package (RRID:SCR_012802; ref. 60) for *in vitro*–treated samples or using the nonparametric NOISeq (RRID:SCR_003002) package (61) for *in vivo*–treated samples presenting lower sequencing depth and more intrinsic variability. Genes with less than 5 counts were filtered out and counts were processed for a trimmed mean of M values (TMM) normalization. DEG was assessed by imposing an FDR <0.05 and a log_2_ fold change cutoff of 1. The pheatmap function was used for data visualization. For k-means clustering, the 2,500 most variable genes were included and six clusters were defined according to the elbow method with the iDEP9.2 online tool, followed by Gene Ontology analysis for Biological Process (http://bioinformatics.sdstate.edu/idep/).

#### Gene Ontology Analysis.

Ontology analyses of differentially expressed genes were performed with the g:Profiler web server (RRID:SCR_006809) for functional profiling (https://biit.cs.ut.ee/gprofiler/gost).

#### sEV Score.

We used public data sets to compare gene-expression profiles of normal B cells and CLL cells in human (GSE67640) and mouse (GSE175564; refs. 16, 21). GSE67640 was analyzed with GEO2R online tool [RRID:SCR_016569; *limma* (RRID:SCR_010943), Benjamini and Hochberg (false discovery rate) adjusted *P* < 0.05, and log_2_FC >1). The DEG lists were compared with a list of 143 genes (combining genes involved in sEV biology based on literature review and Top100 proteins found in sEVs according to http://microvesicles.org, listed in Supplementary Table S2). The expression values of genes present in both lists (shown in the heat maps) were used to compute sEV scores for human and mouse B and CLL cells, corresponding to a z-scored-geometric mean of expression values.

### Proteomics

#### Sample Preparation of sEV Proteome.

Starting from the same amount of sEV proteins between conditions, each sEV sample was split into triplicates. Proteins were extracted with sodium deoxycholate (SDC, 3% final concentration) following a 30-minute incubation at 4°C in the presence of protease inhibitors (cOmplete EDTA-free Protease Inhibitor Cocktail, Roche). Following centrifugation at 16,000 × *g* for 10 minutes, supernatants were subjected to protein reduction (5 mmol/L DTT, 1-hour incubation at 37°C) and alkylation (10 mmol/L IAA, 45 minutes at RT in the dark). Samples were then acidified with formic acid (FA, 1% final concentration), and SDC was precipitated after centrifugation at 16,000 × *g* for 15 minutes. The supernatants were transferred into clean tubes and supplied with preprepared SP3 beads (Fisher Scientific, #09-981-121, #09-981-123). One volume of acetonitrile (ACN, 100% v/v) was added immediately followed by incubation at RT for 10 minutes. The supernatants were removed using a magnetic rack, and the beads were rinsed first with 200 μL of 70% ethanol, and then further rinsed with 180 μL of 100% ACN. Rinsed beads were reconstituted in 30 μL digestion buffer (50 mmol/L ammonium bicarbonate, pH 8). Protein digestion was performed with 1 μg of sequencing grade trypsin (Promega, V5111) for 4 hours at 37°C, then incubated overnight at 37°C with additional 1 μg trypsin. After digestion, ACN was added to each sample to a final concentration of 95%. Mixtures were incubated for 10 minutes at RT and then placed on a magnetic rack for 2 minutes. The supernatants were discarded, and the beads were rinsed with 180 μL of 100% ACN. Rinsed beads were reconstituted in 30 μL LC-MS grade water and incubated for 5 minutes at RT to elute the digested peptides. The eluted peptide samples were acidified with formic acid to a final concentration of 0.1%.

#### Proteomic Sample Preparation of sEV-treated Cells.

CD8^+^ T cells treated with sEVs were collected in triplicate after 24 hours and 4 days of treatment. After being harvested, cells were washed twice with cold PBS. Next, pellets were resuspended in lysis buffer (6 mol/L urea, 2 mol/L thiourea, 50 mmol/L ammonium bicarbonate, pH 8) supplemented with fresh prepared cOmplete EDTA-free Protease Inhibitor Cocktail (Roche). After incubation at 22°C for 30 minutes, samples were sonicated (3-second sonication and 2-second pause for a total of 30 seconds). The supernatants were taken into new tubes following centrifugation at 16,000 × *g* for 15 minutes. Protein quantification assay of the cell extracts was performed with Bradford assay (Sigma-Aldrich, B6916) to estimate the concentrations. A total of 30 μg of each sample were taken for protein reduction and alkylation and digested with Lys-C (FUJIFILM Wako, 125-05061) at 1:30 ratio (enzyme/protein substances) for 4 hours at 37°C, and then samples were diluted 4 times with 50 mmol/L ammonium bicarbonate and digested overnight with 1 μg of trypsin at 37°C. The protein digestion was terminated with the addition of formic acid (1% final concentration). The digested peptides were cleaned up with reverse phase Sep-Pak C18 1 cc Vac Cartridge (Waters, WAT054955) and eluted with 1 mL 50% ACN. Eluted peptides were dried by Speedvac (Thermo Fisher Scientific) and resuspended in 0.1% formic acid. Nanodrop was used to estimate the peptide concentration.

#### LC-MS/MS Data Acquisition.

Digested peptides were measured by LC-MS/MS on either Q-Exactive Plus or Q-Exactive HF mass spectrometer (Thermo Fisher) connected to a Dionex Ultimate 3000 (Thermo Fisher). A total of 400 ng of peptides were loaded onto a trap column (Acclaim PepMap 75 μm × 2 cm, C18, 3 μm) and separated on a 25-cm Acclaim PepMap RSLC column (75 μm × 25 cm, C18, 2 μm) using a 150-minute gradient with a flow rate of 0.3 μL/minute. MS data were acquired in data-dependent mode (DDA). Survey scans of peptide precursors from 375 to 1500 m/z were performed at 70,000 resolution with a 3 × 10^6^ ion count target and the top 12 abundant peaks from the survey scan were selected for fragmentation. Tandem MS was performed by isolation at 1.4 m/z with the quadrupole, HCD fragmentation with a normalized collision energy of 28. The MS2 ion count target was set to 1 × 10^5^, and the max injection time was 45 ms. Only precursors with a charge state of 2 to 7 were sampled for MS2. The dynamic exclusion duration was set to 20 s with a 10 ppm tolerance around the selected precursor and its isotopes.

#### Database Searching and Protein Identification.

All raw data were analyzed with MaxQuant (version 1.6.7.0; RRID:SCR_014485) and searched with Andromeda against the mus musculus database from Uniprot. The minimal peptide length was set to 7 amino acids, and a maximum of 3 missed cleavages were allowed. The search included variable modifications of methionine oxidation and N-terminal acetylation, deamidation (N and Q) and fixed modification of carbamidomethyl cysteine. The “Match between run” was checked within a 1-minute retention time window. Mass tolerance for peptide precursor and fragments were set as 10 ppm and 20 ppm, respectively. The FDR was set to 0.01 for peptide and protein identifications. Label-free quantification was used for quantitative data of identified protein based on its razor and unique peptides. Proteus, an R-package, was used for the downstream analysis of MaxQuant (RRID:SCR_014485) output. The input for Proteus is the evidence file. Evidence data are aggregated into peptides and then into proteins.

#### Proteomic Data Analysis.

For sEV proteome, LFQ intensities of proteins identified in HCME-sEVs, LME-sEVs, and LME-sEV TCL1-RAB27DKOs were processed as follows. We first removed zero intensities across all samples, and then we performed log_2_ transformation and quantile normalization. As samples were acquired in two batches, we performed batch correction using the combat algorithm. Values were then imported in the MaxQuant Perseus software (version 1.6.15.0). Two-sample tests were performed and proteins with a *q*-value < 0.05 (Benjamini–Hochberg FDR) were considered statistically enriched in a condition. Coordinates from PCA were exported and plotted with GraphPad Prism (version 9.1.2; RRID:SCR_002798). Heat maps were generated with the R pheatmap function.

#### Gene and Protein Ontology Analysis.

Ontology analyses of differentially expressed proteins were performed with the g:Profiler (RRID:SCR_006809) web server for functional profiling (https://biit.cs.ut.ee/gprofiler/gost).

### Metabolomics Analysis

#### Detection of Metabolic Protein by Western Blot.

To assess alterations of metabolic protein expression induced by ME-sEV treatment, total proteins isolated from treated CD8^+^ T cells were subjected to western blot: PFKP (#ab204131, RRID:AB_2828009, Abcam), PHGDH (#HPA021241, RRID:AB_1855299, Sigma-Aldrich), PCK2 (#6924S, RRID:AB_10836185, Cell Signaling Technology), MCT4 (#sc-50329, RRID:AB_2189333, Santa Cruz) and GLUT1 (#PA1-46152, RRID:AB_2302087, Thermo Fisher).

#### Stable Isotope Tracing and Metabolite Extraction.

Stable isotope tracing experiments with [U-^13^C]-glucose tracer (Cambridge Isotope Laboratories, CLM-1396) were performed in RPMI-1640 medium supplemented with 11.1 mmol/L [U-^13^C]-glucose, 2 mmol/L glutamine, 10% FBS, 1% P/S, IL2 (10 ng/mL), CD28 (3 μg/mL). For [U-^13^C]-glutamine tracing (Cambridge isotope Laboratories, CLM-1822), RPMI-1640 medium for SILAC (#A24942-01) was used and supplemented as above with the addition of 1.15 mmol/L arginine and 0.219 mmol/L lysine. A total of 1 × 10^6^ cells were seeded in triplicates in 24-well plates precoated with anti-CD3 antibody (10 μg/mL), already in the [U-^13^C]-glucose or glutamine medium. At the time point of metabolite extraction, cell number and volume were determined to calculate packed cell volume. For metabolite extraction, cells were collected and pelleted at 350 × *g* for 5 minutes at 4°C and the medium was stored at −80°C to determine metabolites exchange rates. To determine the basal medium composition for the subsequent calculation of exchange rates, an identical medium was incubated in empty 24 wells throughout the experiment and analyzed in parallel to the medium samples. The cell pellet was then washed with ice-cold 1× PBS solution. 400-μL ice-cold extraction fluid [acetonitrile/H2OMQ/methanol (ratio, 3:2:5); LC-MS grade solvents)] was added to each cell pellet. Cells were mixed for 10 minutes on a thermomixer at 4°C at maximum speed, then the tubes were centrifuged for 10 minutes at 16,100 × *g* at 4°C. 100 μL of the supernatant was collected and transferred to an already-cooled LC-MS glass vial with inserts and stored at −80°C until measurement.

#### YSI Measurements and Medium Exchange Rates.

Medium samples were filtered (PVDF, 0.22 μm) prior to measurement to remove particles. Absolute quantitative values for lactic acid, glutamine, glutamic acid, and glucose were acquired using a YSI 2950D Biochemistry Analyzer (Kreienbaum KWM). For a precise and reliable quantification, external concentration curves of each target compound were prepared and measured in triplicates. Absolute uptake and release rates were calculated as previously described (62).

#### LC-MS Measurements.

The following analytical conditions are based on a previously published protocol (62). Metabolite analyses were performed using a Thermo Vanquish Flex Quaternary LC coupled to a Thermo Q-Exactive HF mass spectrometer. Chromatography was carried out with a SeQuant ZIC-pHILIC 5-μm polymer (150 × 2.1 mm) column connected to the corresponding SeQuant ZIC-pHILIC Guard (20 × 2.1 mm) precolumn. Column temperature was maintained at 45°C. The flow rate was set to 0.2 mL/minute and the mobile phases consisted of 20 mmol/L ammonium carbonate in water, pH 9.2 (Eluent A), and Acetonitrile (Eluent B). The gradient was: 0 minutes, 80% B; 2 minutes, 80% B; 17 minutes, 20% B; 18 minutes 20% B; 19 minutes 80% B; 20 minutes 80% B (0.4 mL/minute); 24 minutes 80% B (0.4 mL/minute); 24.5 minutes 80% B. The injection volume was 5 μL. All MS experiments were performed using electrospray ionization with polarity switching enabled (+ESI/−ESI). The source parameters were applied as follows: sheath gas flow rate, 25; aux gas flow rate, 15; sweep gas flow rate, 0; spray voltage, 4.5 kV (+)/3.5 kV (–); capillary temperature, 325°C; S-lense RF level, 50; aux gas heater temperature, 50°C. The Orbitrap mass analyzer was operated at a resolving power of 30,000 in full-scan mode (scan range, m/z 75…1,000; automatic gain control target: 1e6; maximum injection time: 250 ms). Data were acquired with Thermo Xcalibur software (Version 4.3.73.11; RRID:SCR_014593) and analyzed with TraceFinder (Version 4.1). Subsequent data analysis for normalization and natural isotope subtraction was performed using in-house scripts as previously described (62).

#### OCR Using SeaHorse.

A Seahorse XFe96 Bioanalyser (RRID:SCR_019545, Agilent) was used to determine the basal OCR of CD8^+^ T cells after *ex vivo* treatment with ME-sEVs (96h), following the manufacturer's instructions using WAVE software (RRID:SCR_014526). Treated cells were washed in assay media (XF Base media (Agilent) with glucose (10 mmol/L), sodium pyruvate (1 mmol/L), and L-glutamine (2 mmol/L; Gibco), pH 7.4 at 37°C) before being plated onto Seahorse cell culture plates coated with Cell-Tak (Corning) at 3.5 × 10^5^ cells/well followed by a gentle centrifugation (5 minutes, 300 × *g*, RT, 0 break).

### Statistical Analysis of sEV Gene Expression in CLL Patient Cohort

#### Differential Expression Analysis and Predictive Power.

CT values obtained from real-time PCR were normalized using CTs of ribosomal RNA 28S as a housekeeping gene. Differential expression analysis was performed to check which genes were associated with patient groups by the “limma” package of R/Bioconductor (RRID:SCR_010943; ref. 63).

#### Survival Analysis and Risk Score.

Univariate Cox regression models independently for TFSOS were built. R-package “survival” was used for the analysis and visualization of the data. Normalized log expression of the considered genes was used for the regression model. Individual genes were combined to a risk score with better prognostic properties based on previous publications (64, 65). However, in the current study, we weighted the contribution of each gene by its *P* value: the risk score (RS) for *i*th patient was calculated as




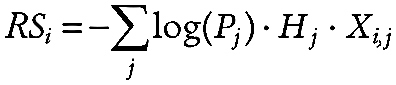




where *P_j_* and *H_j_*—a *P* value of likelihood ratio test and a log HR from a univariate Cox model of the *j*th gene accordingly, *X_i,j_* is the log expression of the *j*th gene in the *i*th patient. Here we run an exhaustive search of all gene combinations and selected the Cox model with the minimal *P*-value. Kaplan–Meier plots were built to visualize the linkage between genes or RS and patient survival. The median level was used to dichotomize patients into two groups. Significance of the variation in RS regarding mutational status and prognosis markers for each patient was assessed by ANOVA.

#### Analysis Using Logistic Regression.

We tried to link gene expression and binary outcomes using logistic regression (“glm” from R/Bioconductor). In order to avoid uncertainty resulting from gene–gene correlation, we used a similar approach as for Cox regression. First, coefficients or log odds ratios and *P* values were determined from univariable logistic models for each gene independently and then combined into a score using the same formula as for the RS in Cox regression, with log odds ratios instead of log-hazard ratios.

### Statistical Analysis

Statistical analysis was performed using GraphPad Prism software (version 9.1.2; RRID:SCR_002798). Data are presented as mean ± standard error of the mean (SEM). The log-rank test was used for the survival curves. For the percentage of CLL cells in mice over time, we performed two-way ANOVA followed by Bonferroni's multiple comparison test. The unpaired t test was used for the rest of the figures. A *P*-value lower than 0.05 was considered statistically significant. Significance displayed in each figure is explained in figure legends.

### Data Availability Statement

The data sets presented in this study are openly available in the Gene-Expression Omnibus with the accession numbers GSE188898, GSE189391, GSE190789, GSE190790, and GSE191186.

## Supplementary Material

Supplementary Methods and Figures Supplementary Methods and Figures

Supplementary Table S1 Supplemental Table S1 - Proteins detected in sEV preparations or cells treated with sEV

Supplementary Table S2 Supplemental Table S2 - Differential expression analysis of genes in leukemic or immune cells treated with sEV

Supplementary Table S3 Supplemental Table S3 - Patient clinical information

Supplementary Table S4 Supplemental Table S4 - Flow cytometry antibody list
