# Supplementary material for: Extracellular Vesicle Secretion by Leukemia Cells In Vivo Promotes CLL Progression by Hampering Antitumor T-cell Responses
Source: Blood Cancer Discov. 2022 Sep 14;4(1):54–77. doi: 10.1158/2643-3230.BCD-22-0029 (PMC9816815; doi:10.1158/2643-3230.BCD-22-0029)
Supplement: Supplementary Methods and Figures [file bcd-22-0029_supplementary_methods_and_figures_suppsf1-sf9.pdf]

Supplemental materials and methods

Plasma-derived sEV isolation

Through cardiac puncture, 1mL of blood/mouse was isolated per group. Using consecutive centrifugations, 400µL of plasma was recovered. Upon filtration (0.22µm), plasma-derived sEV were concentrated and isolated via size exclusion chromatography (SEC) using Exo-spin™ mini (#EX03-25, Cell Guidance System) following the manufacturer's instructions (**Fig.S1E**).

Analysis of spleen architecture and cellularity

Spleens from age-matched C57BL/6, leukemic TCL1, and TCL1-RAB27DKO were fixed for 24h in 10% neutral buffered formalin v/v (Simport). The spleens were then washed in 70% Ethanol, embedded in paraffin, cut at 3µm thickness and HE stained according to standard protocols. The histological slides were evaluated by an experienced hematopathologist (JAF).

Transfection of miRNA Mimics into HCME-sEV

Transfection of miRCURY LNA™ miRNA Mimics of miR-150 (MMU-MIR-150-5P, # 339173 YM00470312-ADA, Qiagen), -155 (MMU-MIR-155-5P, # 339173 YM00472490-ADA, Qiagen) and -378a (MMU-MIR-378A-5P, # 339173 YM00470442-ADA, Qiagen) or scramble control (Negative control, #339173 YM00479902-ADA, Qiagen) into HCME-sEV was performed using HiPerFect® Transfection Reagent (#301704, Qiagen) following a protocol adapted from Wang *et al.*[1]. Briefly, 20 pmol of miRCURY LNA™ miRNA Mimics were diluted in medium without serum, 2uL transfection reagent were added, and mixed by vortexing. The formation of molecular complexes was allowed for 10 min at RT. Next, the complexes were added drop-wise onto HCME-sEV and incubated at 37°C for 6h. sEV were washed once in PBS before adding them to CD8+ T-cells for 48h.

$CD8^+$ T-cell Proliferation Assay

A total of $5{\times}10^5$ freshly isolated $CD8^+$ T-cells were labeled Carboxyfluorescein N hydroxysuccinimidyl ester (CFSE) at a concentration of 1µM for 5 min. Medium containing LME-sEV treated cells was provided with or without 10µM of ATP. Three days later, the cells were harvested and analyzed by flow cytometry.

Transcriptomics

Microarrays

The Affymetrix microarray chips "Clariom™ S Assay, mouse" (Applied Biosystems™) were used following manufacturer's instructions described in the GeneChip® 3' IVT Pico Reagent Kit guide. Briefly, 1ng total RNA was used for cRNA synthesis. cRNA was converted to cDNA and 4µg ss-cDNA were hybridized on Clariom™ S Assay, mouse arrays. Raw CEL files were analyzed with the Transcriptome Analysis Console TAC 4.0.

Transcription factor enrichment analysis

Fastq files were submitted to the free online tool ISMARA (Integrated System for Motif Activity Response Analysis, https://ismara.unibas.ch/mara/) to perform a transcription factor enrichment analysis through the recognition of most important transcription factors that are impacting on transcription profiles of a set of samples.

Proteomics

Gene-miRNA Interaction network

The list of proteins down-regulated in $CD8^+$ T-cells incubated with LME-sEV *in vitro* for 4 days was submitted to the ShinyGO online tool (version 0.66, http://bioinformatics.sdstate.edu/go/) and an enrichment analysis was performed against the miRNA.Target.DIANATarBase gene set. All gene-miRNA interactions with FDR < 0.1 were exported. Predicted interactions for miR-150-5p, miR-378a-3p, miR-146a-5p, miR-155-5p, and miR-21a-5p were imported to Cytoscape (v3.8.2; RRID:SCR_003032) to build the interaction network.

Supplemental References

1.Wang F, Li L, Piontek K, Sakaguchi M, Selaru FM. Exosome miR-335 as a novel therapeutic strategy in hepatocellular carcinoma. *Hepatology*. 2018;67(3):940-954.

2.Haderk F, Schulz R, Iskar M, et al. Tumor-derived exosomes modulate PD-L1 expression in monocytes. *Sci Immunol*. 2017;2(13).

Supplemental Figure S1, related to Figure 1

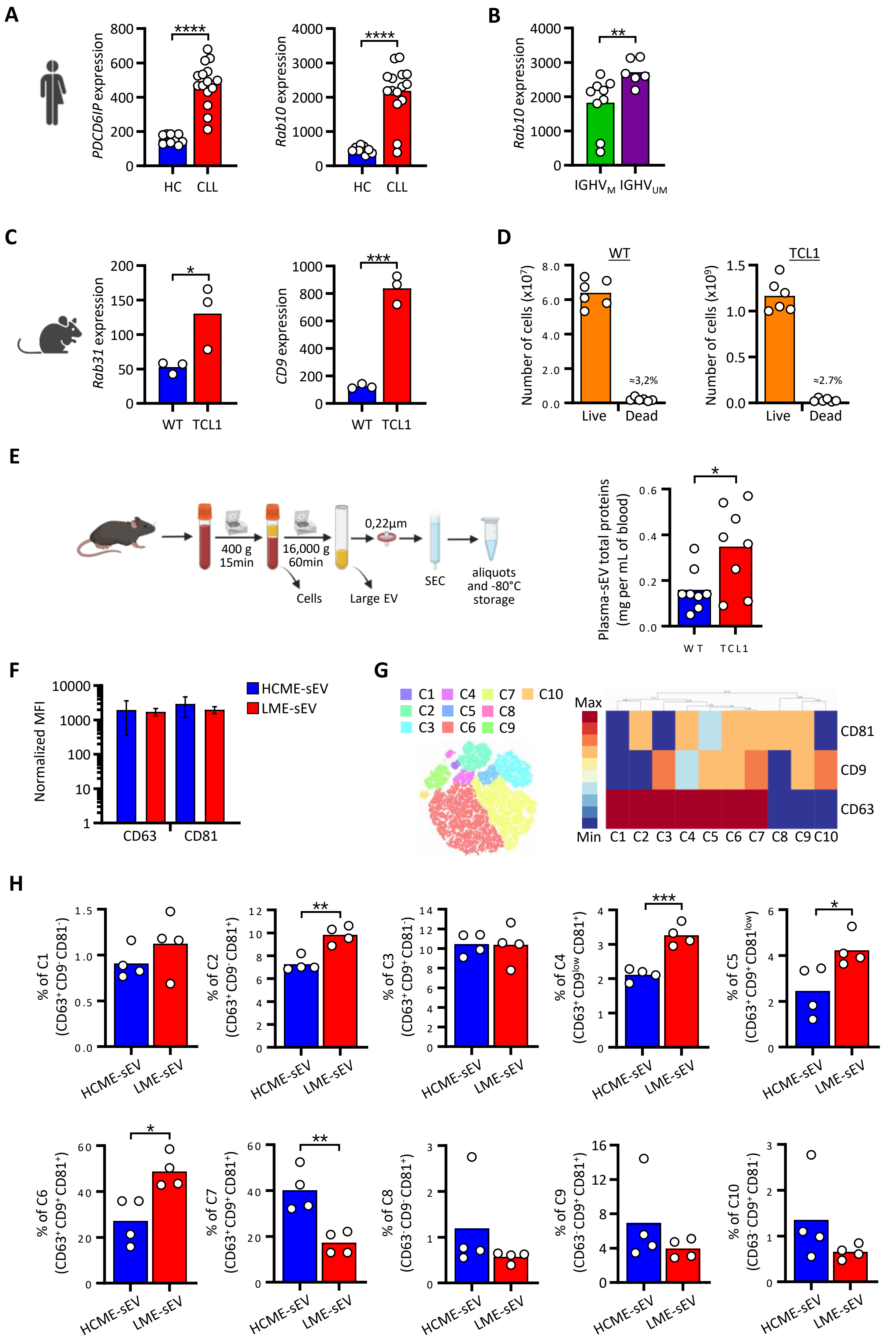

Supplemental Figure S1: Small EV are enriched in leukemic microenvironment. Related to Figure 1
(A) mRNA levels of selected genes extracted from Figure 1A. **(B)** mRNA expression of *Rab10* according to IGHV mutational status. **(C)** mRNA levels of selected genes extracted from Figure 1F. **(D)** Number of live/dead cells recovered per spleen after dissociation (trypan blue counting). **(E)** Detailed protocol to isolate and purify sEV from PB and relative amount of proteins (in mg) recovered from WT- (n=8) or TCL1-sEV (n=8), normalized per mL of blood. **(F)** Intensity of CD63 and CD81 measured by bead-based FC on LME-sEV (n= 8) or HCME-sEV (n=2, isolated from independent pools of 5 C57BL/6 spleens). **(G)** HSNE clustering analysis of $MB488^{+}$ LME-sEV based on CD63, CD81 and CD9 expression measured by bead-free FC. HSNE clustering depicting ME-sEV origin, cluster identity and marker expression. **(H)** Percentages of ME-sEV subpopulation distribution based on CD63, CD81 and CD9 cluster analysis of panel G. *$p<0.05$, **$p<0.01$, ***$p<0.001$, ****$p<0.0001$ (unpaired Student *t* test). Data are mean.

Supplemental Figure S2, related to Figure 2

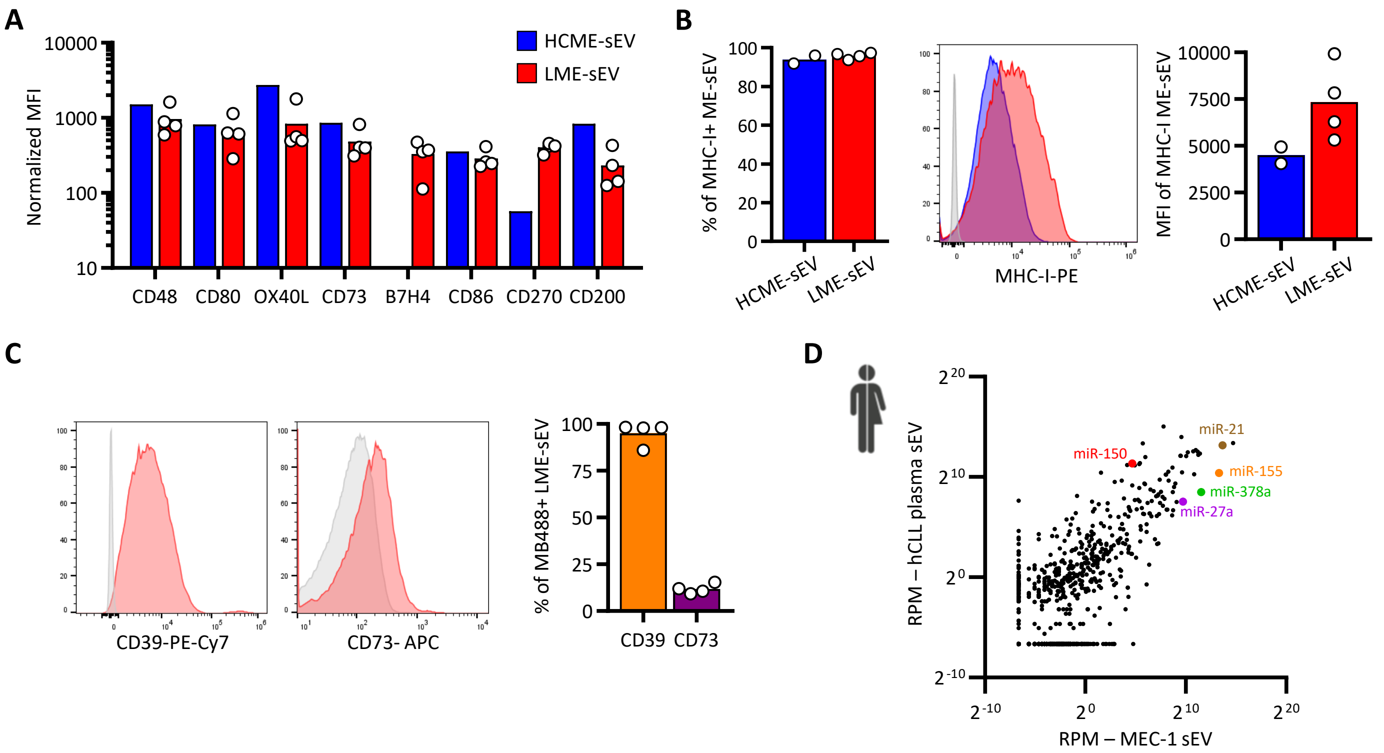

Supplemental Figure S2: LME-sEV present a specific proteome and miRNA fingerprint. Related to Figure 2

(A) Expression of ICP ligands on HCME- or LME-sEV quantified by bead-based FC. **(B)** Surface expression of MHC-I on single HCME- and LME-sEV analyzed by FC and relative quantification. **(C)** Surface expression of CD39 and CD73 on single LME-sEV analyzed by FC and relative quantification. **(D)** Correlation in miRNA composition between CLL patient plasma- and MEC-1-derived sEV based on gene expression analysis (RPM = reads per million, data from Haderk et al[2]). Data are mean.

Supplemental Figure S3, related to Figure 3

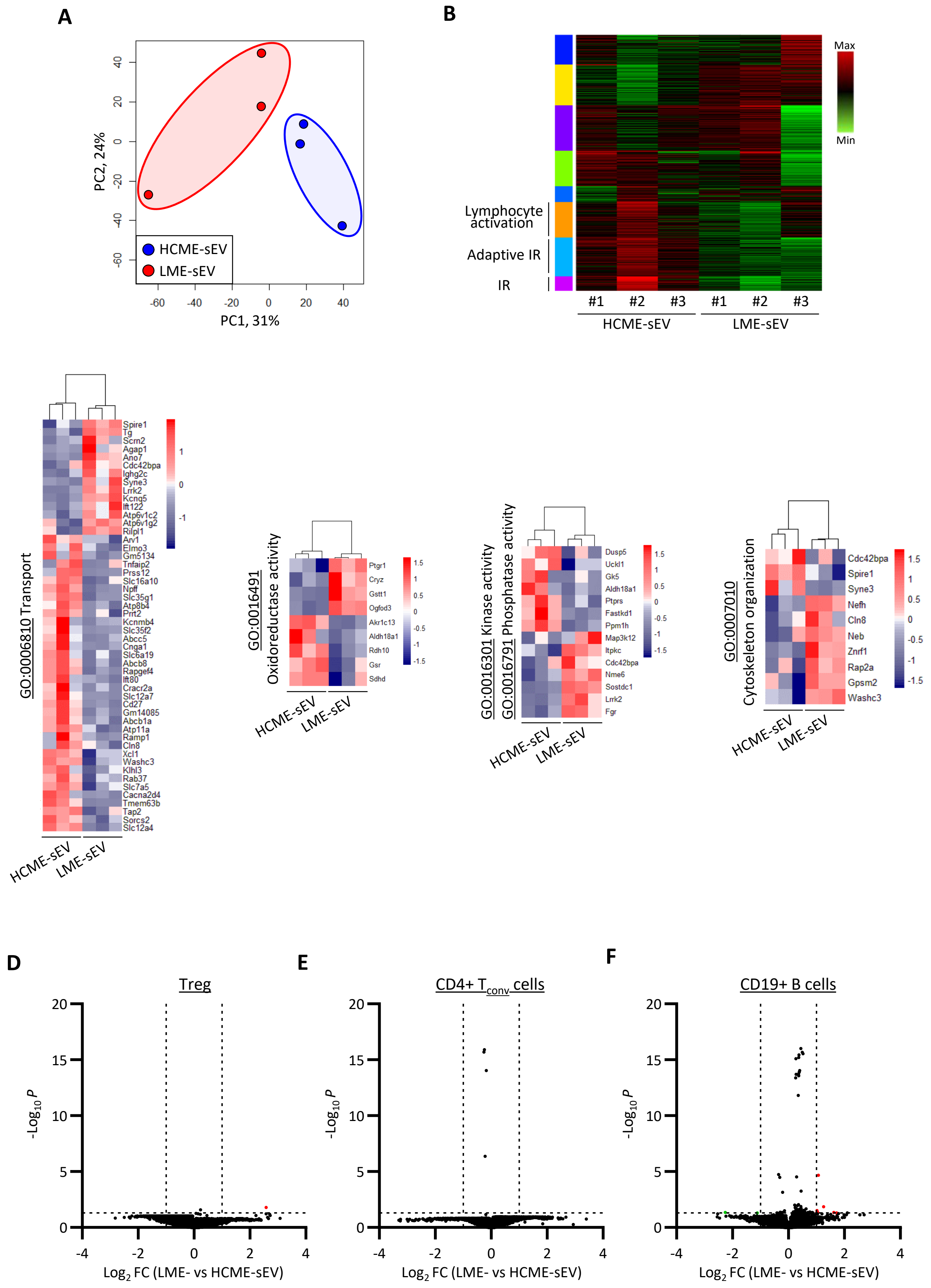

Supplemental Figure S3: Effect of LME-sEV on lymphocyte subsets in the microenvironment. Related to Figure 3

(A) PCA of $CD8^+$ T-cells isolated from spleens of mice treated with LME- or HCME-sEV for 1 week (from Figure 3I). **(B)** k-means clustering of the 2,500 most variables genes from Figure 3I followed by gene ontology analysis (IR: immune response). **(C)** Hierarchical clustering of selected genes from Figure 3J, grouped by enriched gene ontologies. **(D-F)** Volcano plots showing the absence of DEG measured by RNA-seq with FDR < 0.05 and log2FC >1 in Treg (D) $CD4^+$ T-cells (E) and $CD19^+$ B-cells (F) isolated from spleens of mice treated with LME- or HCME-sEV for 1 week.

Supplemental Figure S4, related to Figure 4

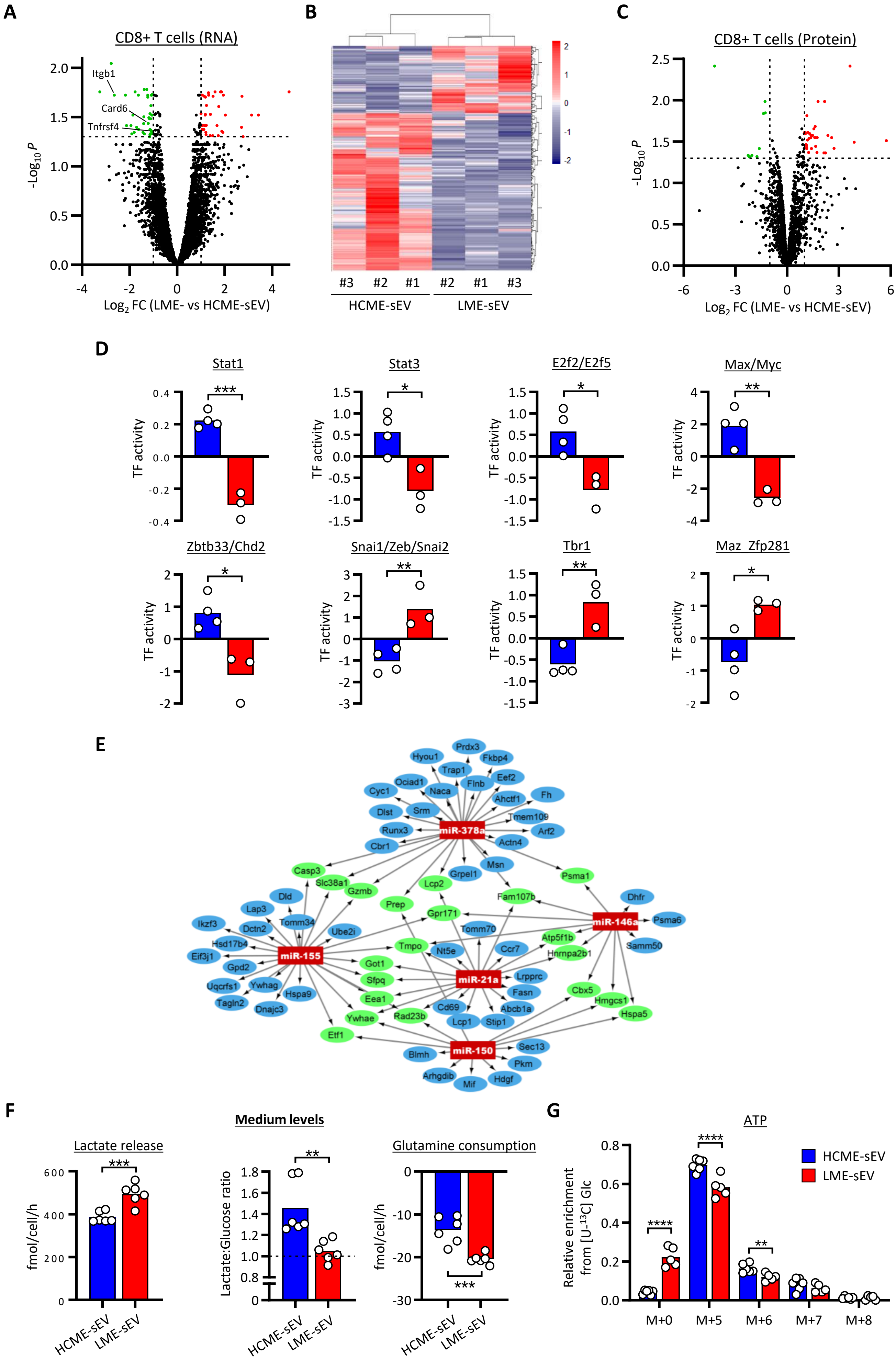

Supplemental Figure S4, related to Figure 4

H

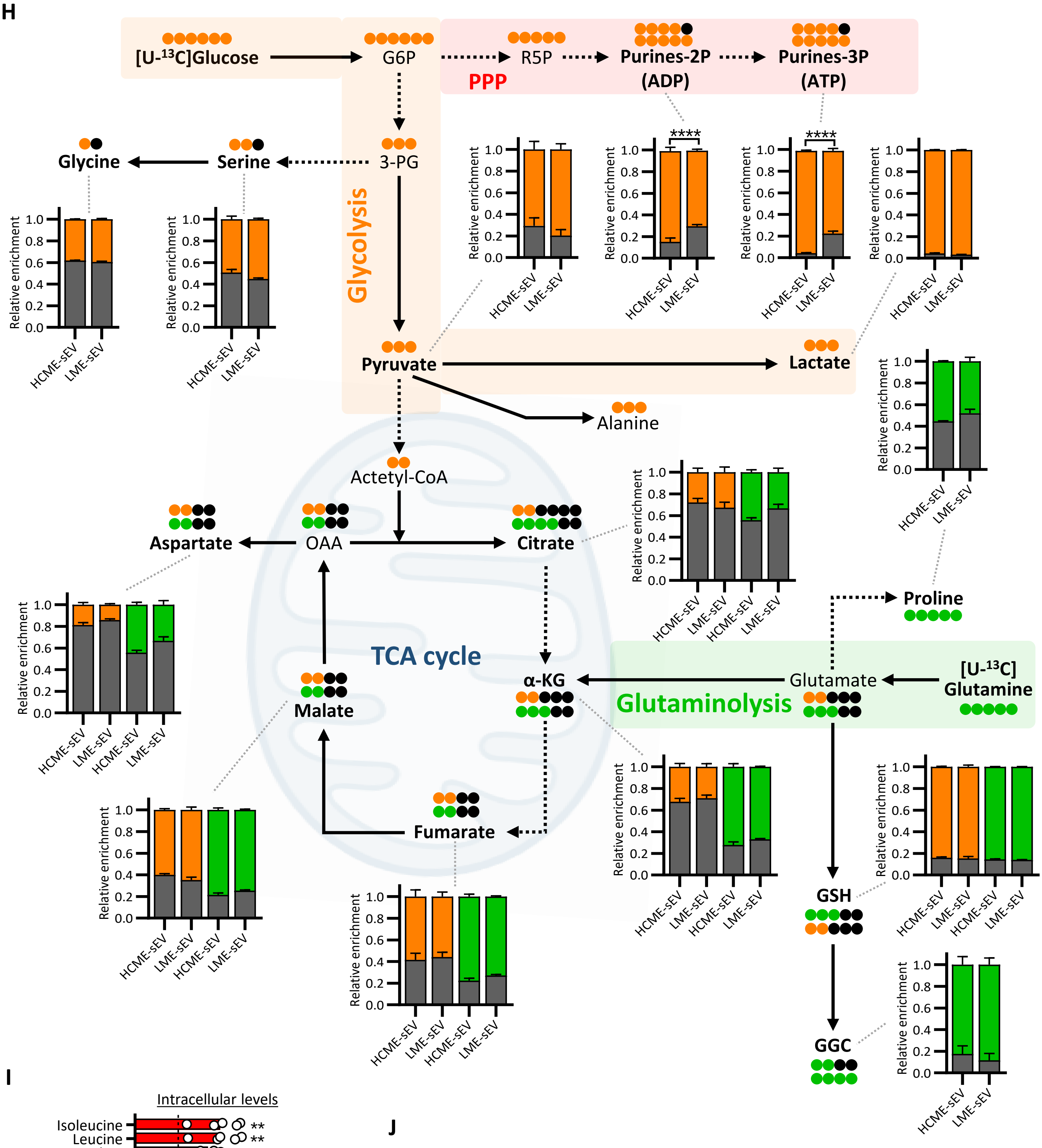

I

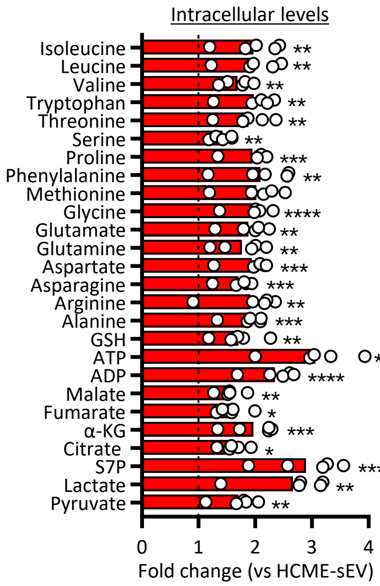

J

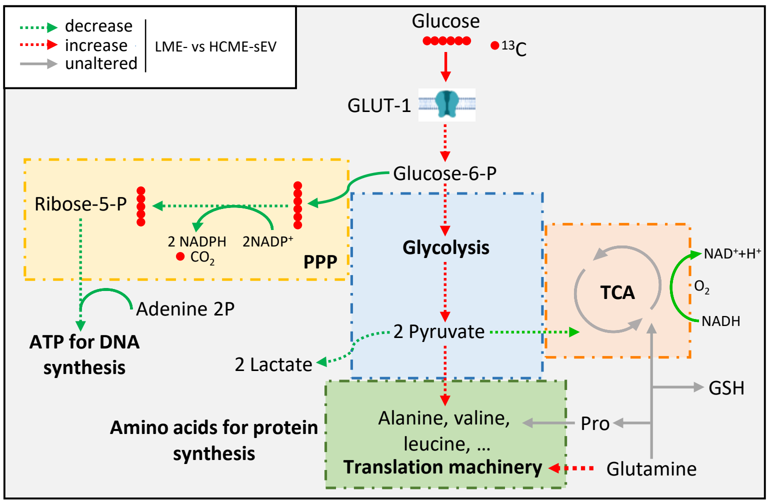

Supplemental Figure S4. LME-sEV alter T-cell subsets. Related to Figure 4

(A) Activity of selected transcription factors from $CD8^+$ T-cells treated with LME- or HCME-sEV (based on Figure 4A). **(B)** Volcano plot showing DEG quantified by microarray between $CD8^+$ T-cells treated for 24h with LME- (n=3) and HCME (n=3) with FDR<0.05 and $log_2FC>1$. **(C)** Hierarchical clustering of DEG from panel B. **(D)** Volcano plot showing DEP determined by mass spectrometry between $CD8^+$ T-cells treated for 24h with LME- (n=3) and HCME-sEV (n=3) with FDR<0.05 and $log_2FC>1$. **(E)** Interaction network miRNA-proteins depicting target prediction based on less abundant DEP (from Figure 4D), and highlighting the miRNA enriched in LME-sEV (from Figure 2J). Blue circle represent proteins targeted by one miRNA while proteins in green circles are targets of multiple miRNAs. **(F)** Levels of lactate, lactate:glucose ratio and glutamine measured by mass spectrometry in culture medium after treatment of $CD8^+$ T-cells for 96h with LME- or HMCE-sEV. Negative value represent consumption while positive value represent release. **(G)** Isotopologue analysis by mass spectrometry of ATP produced from ^{13}C-glucose from Fig.4K. **(H)** Overview of metabolite levels determined by mass spectrometry generated from ^{13}C-glucose (orange) and ^{13}C-glutamine (green) in $CD8^+$ T-cells treated with LME- or HMCE-sEV for 96h. **(I)** Fold changes in intracellular levels of metabolites detected by mass spectrometry in $CD8^+$ T-cells treated with LME- or HMCE-sEV for 96h. **(J)** Overall model of metabolic rewiring in $CD8^+$ T-cells treated with LME- vs HCME-sEV (green line: decrease, red line: increase, grey line: unaltered). *p<0.05, **p<0.01, ***p<0.001, ****p<0.0001 (Unpaired Student *t* test). Data are mean and SEM.

Supplemental Figure S5, related to Figure 4

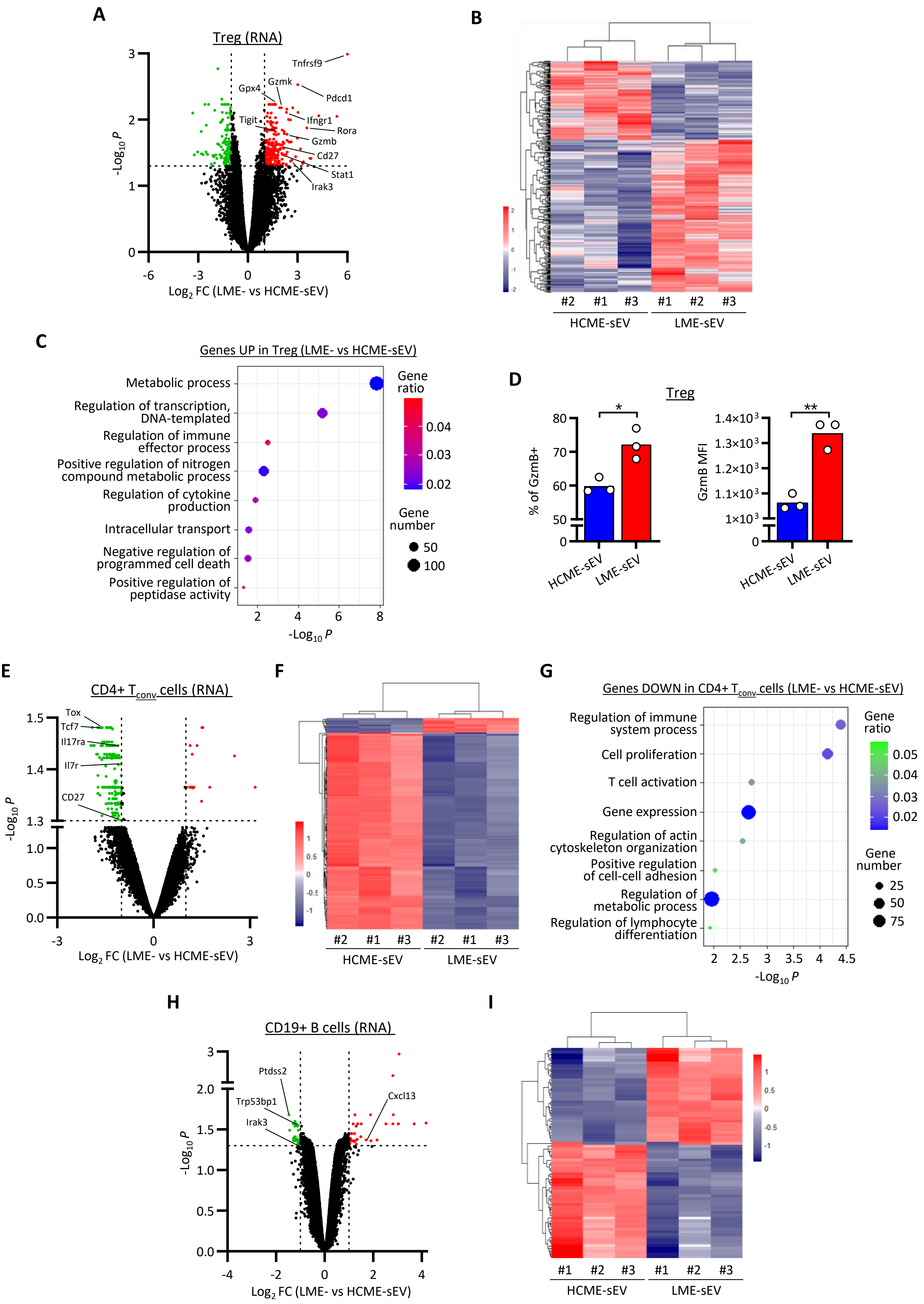

Supplemental Figure S5. LME-sEV alter T-cell subsets. Related to Figure 4

(A) Volcano plot showing DEG determined by microarray in Tregs (YFP$^+$) treated for 24h with LME- (n=3) and HCME-sEV (n=3) with FDR<0.05 and $\log_2$FC>1. **(B)** Hierarchical clustering of DEG from panel A. **(C)** Ontology of enriched DEG from Treg treated for 24h with LME- or HMCE-sEV from Panel A. **(D)** Percentage of Treg expressing GzmB (left panel) and relative intensity (right panel). **(E)** Volcano plot showing DEG determined by microarray between CD4$^+$ T_{conv}-cells treated for 24h with LME- (n=3) and HCME-sEV (n=3) with FDR<0.05 and $\log_2$FC>1. **(F)** Hierarchical clustering of DEG from panel E. **(G)** Ontology of diminished DEG from panel E. **(H)** Volcano plot showing DEG determined by microarray between CD19$^+$ B-cells treated for 24h with LME- (n=3) and HCME (n=3) with FDR<0.05 and $\log_2$FC>1. **(I)** Hierarchical clustering of DEG from panel H. *p<0.05, **p<0.01 (Unpaired Student *t* test). Data are mean.

Supplemental Figure S6, related to Figure 5

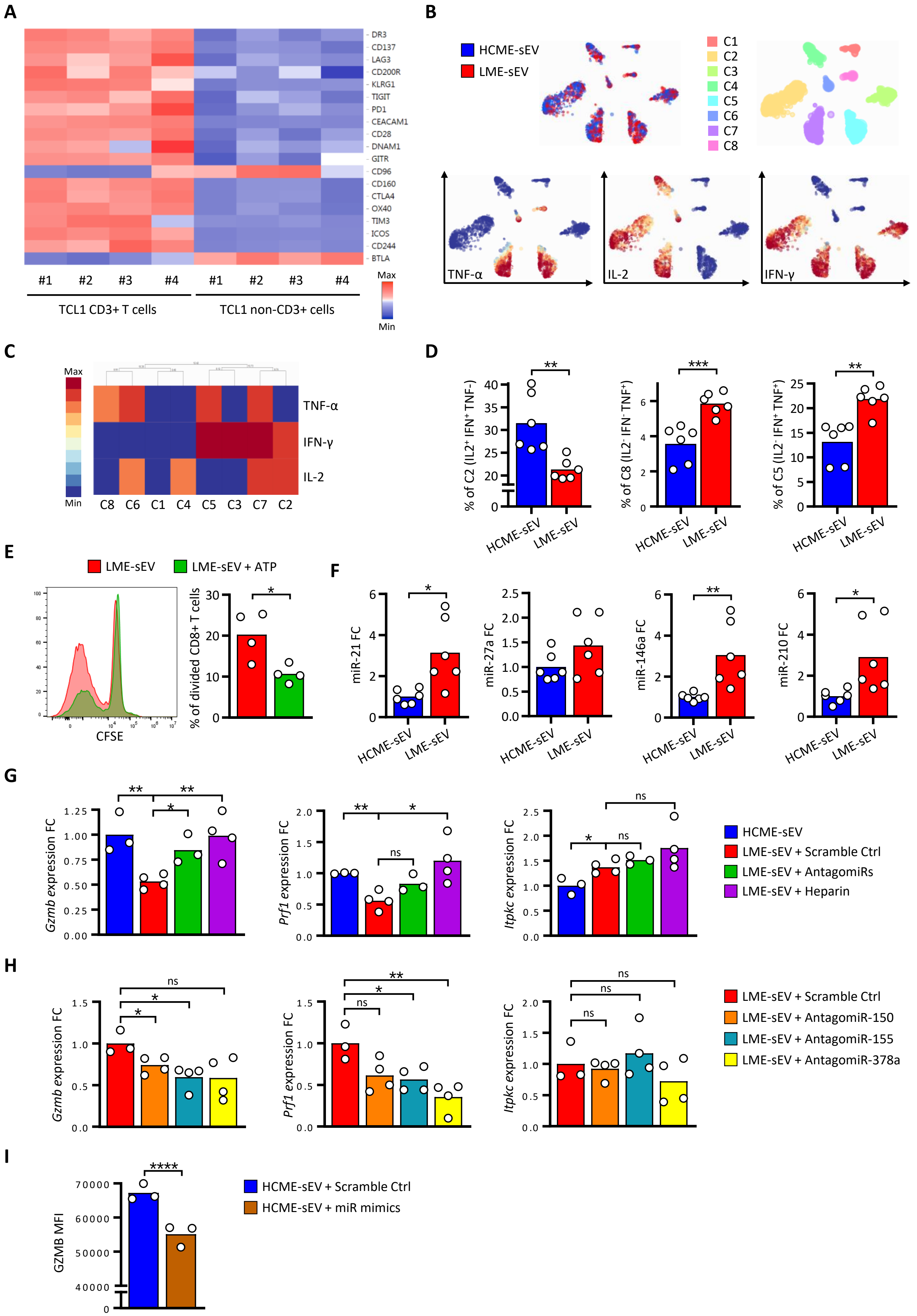

Supplemental Figure S6: LME-sEV decrease CD8$^+$ T-cell functions. Related to Figure 5

(A) ICP receptor levels determined by FC (MFI) on CD3$^+$ T-cells and non-CD3$^+$ cells derived from TCL1 leukemic spleens. **(B)** Cytokine expression measured by FC in CD8$^+$ T-cells treated for 48h with LME- and HCME-sEV. HSNE clustering depicting treatments, cluster identity, and marker expression. **(C)** Hierarchical clustering of clusters based on cytokine expression. **(D)** Percentage of polyfunctional CD8$^+$ T-cells producing combinations of cytokines (Clusters 2, 5 and 8). **(E)** Histogram plot showing division of CFSE-loaded CD8$^+$ T-cells treated with LME-sEV or LME-sEV + ATP (left panel) measured by FC and corresponding percentage (right panel). **(F)** miRNA levels measured by RT-qPCR in CD8$^+$ T-cells treated with HCME- or LME-sEV for 24h. **(G-H)** mRNA expression measured by RT-qPCR of selected genes in CD8$^+$ T-cells treated for 48h with HCME-sEV or LME-sEV transfected with scramble or multiple antagomiRs (miR-150, -155 and -378a) (G) or with single antagomiRs (H). Preincubation of LME-sEV with heparin was used as inhibitor of sEV internalization. **(I)** GzmB level measured with FC in CD8$^+$ T-cells treated for 48h with HCME-sEV transfected with scramble or miRNA mimics (miR-150, -155 and -378a). *p<0.05, **p<0.01, ***p<0.001, ****p<0.0001 (Unpaired Student *t* test). Data are mean.

Supplemental Figure S7, related to Figure 6

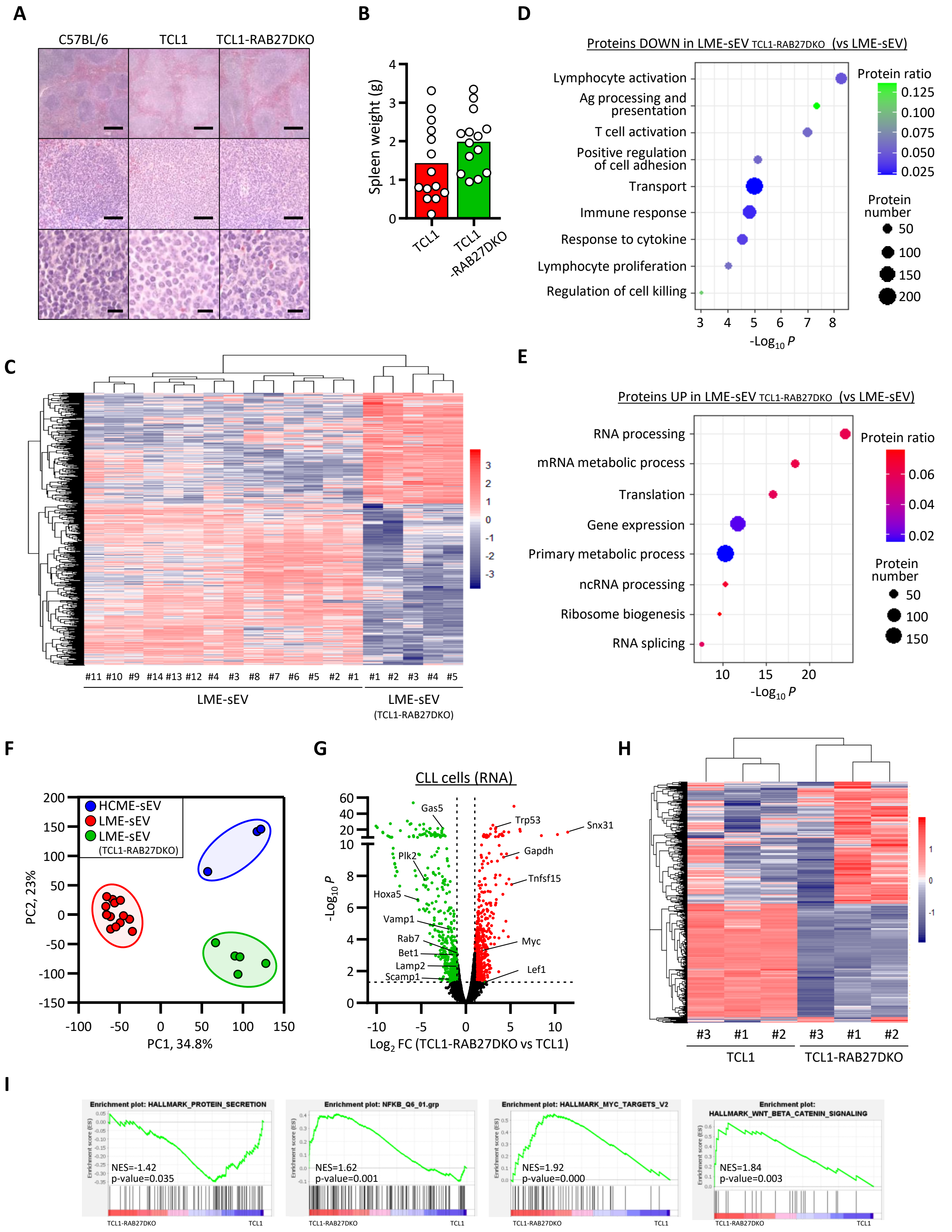

Supplemental Figure S7: Small EV are crucial for CLL development *in vivo.* Related to Figure 6

(A) Histological analyses (H&E staining) performed on C57BL/6, TCL1, and TCL1-RAB27DKO spleens. Scale bars: 500µm (up), 100µm (middle) and 50µm (down). **(B)** Spleen weights of TCL1 and TCL1-RAB27DKO mice at euthanasia (from Figure 6E). **(C)** Hierarchical clustering of DEP determined by mass spectrometry between LME-$sEV_{TCL1-RAB27DKO}$ (n=5) and LME-sEV (n=14), based on Figure 6H. **(D-E)** Protein ontology of DEP in LME-$sEV_{TCL1-RAB27DKO}$ (vs LME-sEV). **(F)** PCA based on DEP between HCME-sEV (n=3), LME-sEV (n=18) and LME-$sEV_{TCL1-RAB27DKO}$ (n=5) (identified by one-way ANOVA, FDR<0.05). **(G-H)** Volcano plot (G) and heatmap (H) showing DEG determined by RNA-seq between $CD5^+CD19^+$ leukemic cells from TCL1-RAB27DKO and TCL1 mice with FDR<0.05 and $log_2FC>1$. **(I)** Plots from preranked Gene Set Enrichment Analysis indicating a decrease in protein secretion and the enrichment in gene sets driven by the transcription factors NF-κB, Myc, and Wnt/β-catenin in TCL1-RAB27DKO cells compared to TCL1 cells. Data are mean.

Supplemental Figure S8, related to Figure 6

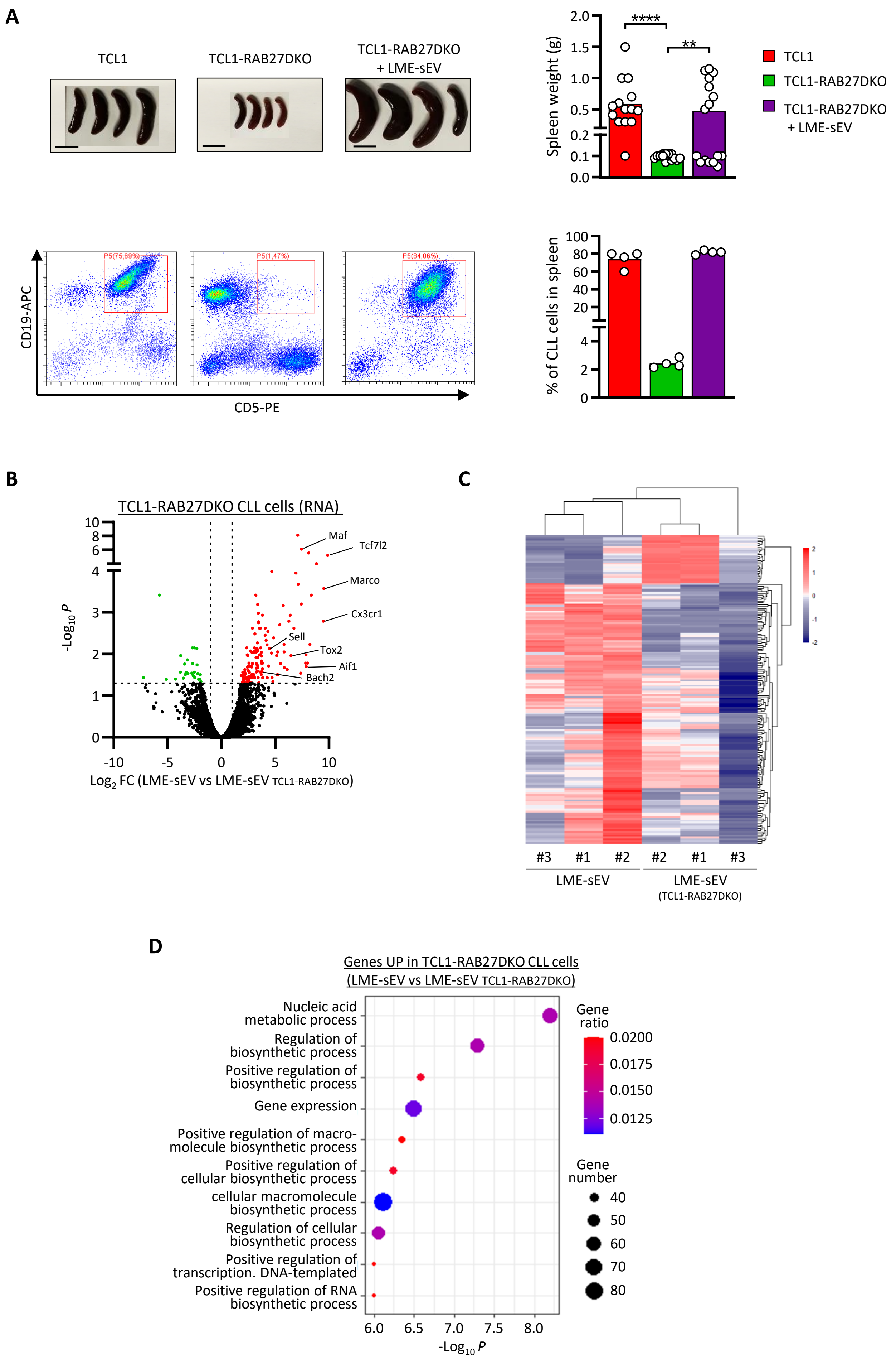

Supplemental Figure S8, related to Figure 6

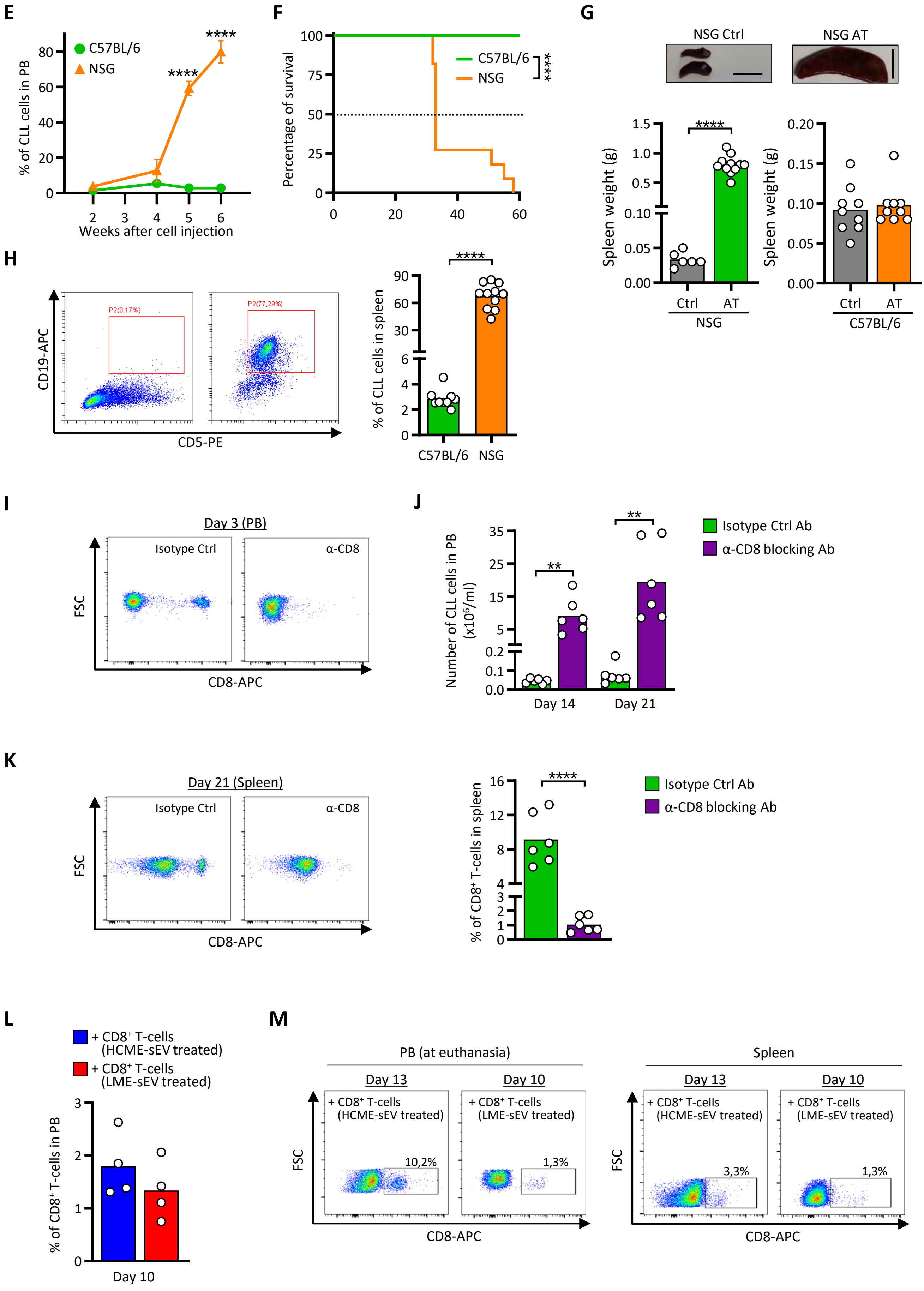

Supplemental Figure S8: Small EV impair the anti-tumor immune response against CLL *in vivo.* Related to Figure 6

(A) Representative pictures of four spleens per group (one per clone, scale bars: 1cm, upper left panel) and spleen weight at euthanasia from all mice of Figure 6J (upper right panel). FC dot plots showing $CD5^+CD19^+$ CLL cells in total splenocytes (lower left panel) and corresponding quantification for four animals per group (one per clone, lower panel). **(B-D)** Volcano plot (B) and heatmap (C) showing DEG determined by RNA-seq in $CD5^+CD19^+$ leukemic cells from TCL1-RAB27DKO treated with LME-sEV from TCL1 or TCL1-RAB27DKO with FDR<0.05 and $\log_2FC>1$ and associated Gene Ontology (D). **(E)** Percentage of $CD5^+CD19^+$ CLL cells determined by FC in peripheral blood (PB) of NSG (n=12) or C57BL/6 (n=9) mice over time. Three different TCL1-RAB27DKO clones were injected each in four NSG and three C57BL/6 mice. **(F)** Survival of mice from panel E. **(G)** Representative pictures of spleens from NSG mice (upper panel, scale bar: 1cm) and spleen weights at euthanasia of NSG and C57BL/6 mice injected with TCL1-RAB27DKO cells (AT) and corresponding healthy controls (Ctrl) (lower panel). **(H)** Representative FC dot plots of $CD5^+CD19^+$ CLL cells in total splenocytes and percentage of $CD5^+CD19^+$ CLL cells in the spleen from panel E at euthanasia. **(I)** Representative FC dot plots of $CD8^+$ T-cell depletion in the PB of mice at day 3 (based on Figure 6K). **(J)** Number of $CD5^+CD19^+$ CLL cells determined by FC in the PB of mice from Figure 6L. **(K)** Representative FC dot plots of $CD8^+$ T-cells in total splenocytes (left panel) and relative percentage (right panel) in the spleen of mice from Figure 6L at euthanasia. **(L)** Percentage of $CD8^+$ T-cells determined by FC in the PB of mice from Figure 6N. **(M)** Representative FC dot plots of $CD8^+$ T-cells in PB (left panel) and total splenocytes (right panel) from Figure 6O at euthanasia. **p<0.01, ****p<0.0001 (unpaired Student *t* test for A, G, H, and J-L, 2-way ANOVA followed by Bonferroni's multiple comparison test for E, log-Rank test for F). Data are mean with SEM.

Supplemental Figure S9, related to Figure 7

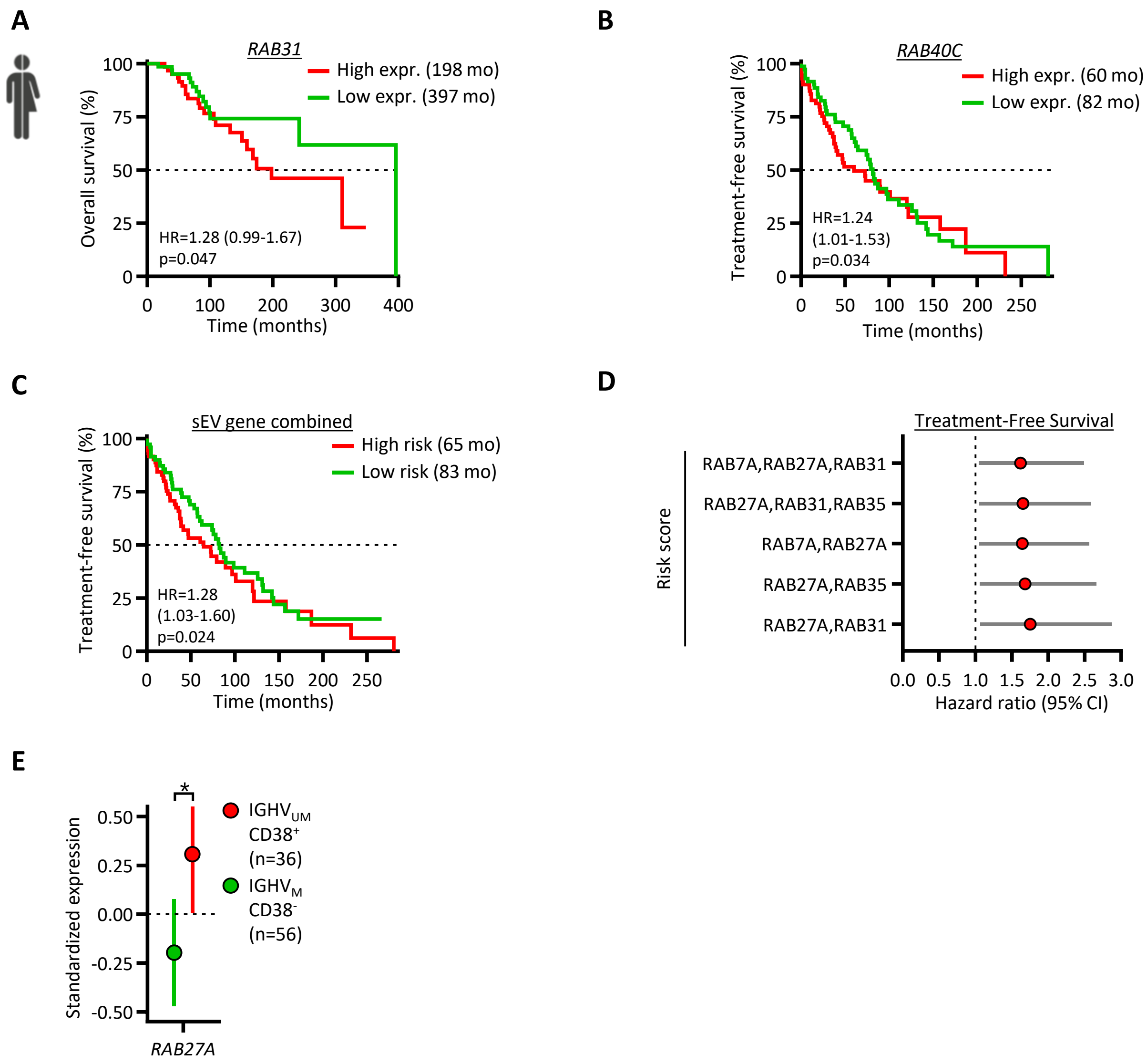

Supplemental Figure S9: Expression of sEV-related genes correlates with disease progression and poor survival in CLL patients. Related to Figure 7.

(A-B) Correlation between high or low single gene expression and OS (A) or TFS (B). Low and High groups are of identical size (n=72). Median survival is indicated in months (mo). **(C)** Correlation between high or low combined 7-gene expression and TFS. **(D)** Calculated hazard ratios >1 (red dots, p-value <0.05) indicate an increased risk for patients with high multiple gene expression in term of TFS**.** **(E)** Expression of *RAB27A* in sub-groups of patients according to prognostic markers (IGHV and CD38, group size indicated in each panel). *p<0.05, **p<0.01. Data are mean with 95% confidence intervals.
